# High prevalence of *APOA1/C3/A4/A5* alterations in luminal breast cancers among young women in East Asia

**DOI:** 10.1038/s41523-021-00299-5

**Published:** 2021-07-05

**Authors:** Ching-Hung Lin, Ruby Yun-Ju Huang, Tzu-Pin Lu, Kuan-Ting Kuo, Ko-Yun Lo, Ching-Hsuan Chen, I-Chun Chen, Yen-Shen Lu, Eric Y. Chuang, Jean Paul Thiery, Chiun-Sheng Huang, Ann-Lii Cheng

**Affiliations:** 1 Department of Oncology, National Taiwan University Hospital, Taipei, Taiwan; 2 Department of Medical Oncology, National Taiwan University Cancer Center Hospital, Taipei, Taiwan; 3 School of Medicine, College of Medicine, National Taiwan University, Taipei, Taiwan; 4 Graduate Institute of Oncology, College of Medicine, National Taiwan University, Taipei, Taiwan; 5 Department of Obstetrics & Gynaecology, Yong Loo Lin School of Medicine, National University of Singapore, Singapore, Singapore; 6 Institute of Epidemiology and Preventive Medicine, Department of Public Health, National Taiwan University, Taipei, Taiwan; 7 Department of Pathology, National Taiwan University Hospital, Taipei, Taiwan; 8 Genomics Research Center, Academia Sinica, Taipei, Taiwan; 9 Department of Obstetrics and Gynecology, Taipei City Hospital Heping Fuyou Branch, Taipei, Taiwan; 10 Graduate Institute of Biomedical Electronics and Bioinformatics and Department of Electrical Engineering, National Taiwan University, Taipei, Taiwan; 11 Bioinformatics and Biostatistics Core, Center of Genomic Medicine, National Taiwan University, Taipei, Taiwan; 12 Institute of Molecular and Cell Biology, A*STAR, Singapore, Singapore; 13 Department of Biochemistry, Yong Loo Lin School of Medicine, National University of Singapore, Singapore, Singapore; 14 Department of Surgery, National Taiwan University Hospital, Taipei, Taiwan; 15 Graduate Institute of Oncology and Cancer Research Centre, College of Medicine, National Taiwan University, Taipei, Taiwan

**Keywords:** Breast cancer, Cancer genomics

## Abstract

In East Asia, the breast cancer incidence rate among women aged <50 years has rapidly increased. Emerging tumors are distinctly characterized by a high prevalence of estrogen receptor (ER)–positive/human epidermal growth factor receptor (HER2)–negative cancer. In the present study, we identified unique genetic alterations in these emerging tumors. We analyzed gene copy number variations (CNVs) in breast tumors from 120 Taiwanese patients, and obtained public datasets of CNV and gene expression (GE). The data regarding CNV and GE were separately compared between East Asian and Western patients, and the overlapping genes identified in the comparisons were explored to identify the gene–gene interaction networks. In the age <50 years/ER + /HER2– subgroup, tumors of East Asian patients exhibited a higher frequency of copy number loss in *APOA1/C3/A4/A5*, a lipid-metabolizing gene cluster (33 vs. 10%, *P* < .001) and lower *APOA1/C3/A4/A5* expressions than tumors of Western patients. These copy number loss related– and GE–related results were validated in another Taiwanese cohort and in two GE datasets, respectively. The copy number loss was significantly associated with poor survival among Western patients, but not among East Asian patients. Lower *APOA1*, *APOC3*, and *APOA5* expressions were associated with higher ESTIMATE immune scores, indicating an abundance of tumor-infiltrating immune cells. In conclusion, *APOA1/C3/A4/A5* copy number loss was more prevalent in luminal breast tumors among East Asian women aged <50 years, and its immunomodulatory effect on the tumor microenvironment possibly plays various roles in the tumor biology of East Asian patients.

## Introduction

In East Asia countries, the incidence of breast cancer is generally lower than that in Western countries.^1^ Statistics gathered from breast cancer registries in East Asian countries such as Singapore, South Korea, Japan, China, and Taiwan, have indicated that the incidence has been rapidly increasing over the past three decades. The rapid surge of breast cancer incidence in East Asia has been particularly observed in women aged <50 years of age. Some studies cited the stronger birth cohort effect in East Asia as an explanation for this trend.^2–6^ A “Westernized” lifestyle has been thought to be the major cause of the rapid increase of breast cancer among young women in East Asia.^7^

The tumor biology of breast cancer in East Asian young women was considered to be similar to that of Western young women.^8^ However, some studies have revealed distinct differences in clinicopathological features, molecular subtypes, and breast cancer prognosis between young East Asian and young Western women. Specifically, breast cancer in women aged <50 years in East Asia is characterized by a high prevalence of low histological grade and luminal subtype (defined by estrogen receptor [ER] or progesterone receptor [PR]–positive/human epidermal growth factor receptor 2 (HER2)-negative status; ER+/HER2−status) and a favorable prognosis.^9–13^ Our recent study demonstrated the contrasting age-specific incidences and pathological characteristics of breast cancer between East Asian and American women, as well as between East Asian Americans and white Americans, and suggests racial differences in biology.^12^ Immigration studies have suggested that the East Asian ancestry effect might contribute to the phenomenon of preferential survival benefit. Data from the Surveillance, Epidemiology, and End Results (SEER) databases, SEER9 and SEER18, indicated that Asian American patients with breast cancer survived longer compared with other ethnic groups, including a group comprising non-Hispanic people of European descent.^14,15^ Therefore, an interaction effect of the birth cohort effect with the genetic background may be present.

**Fig. 1:**
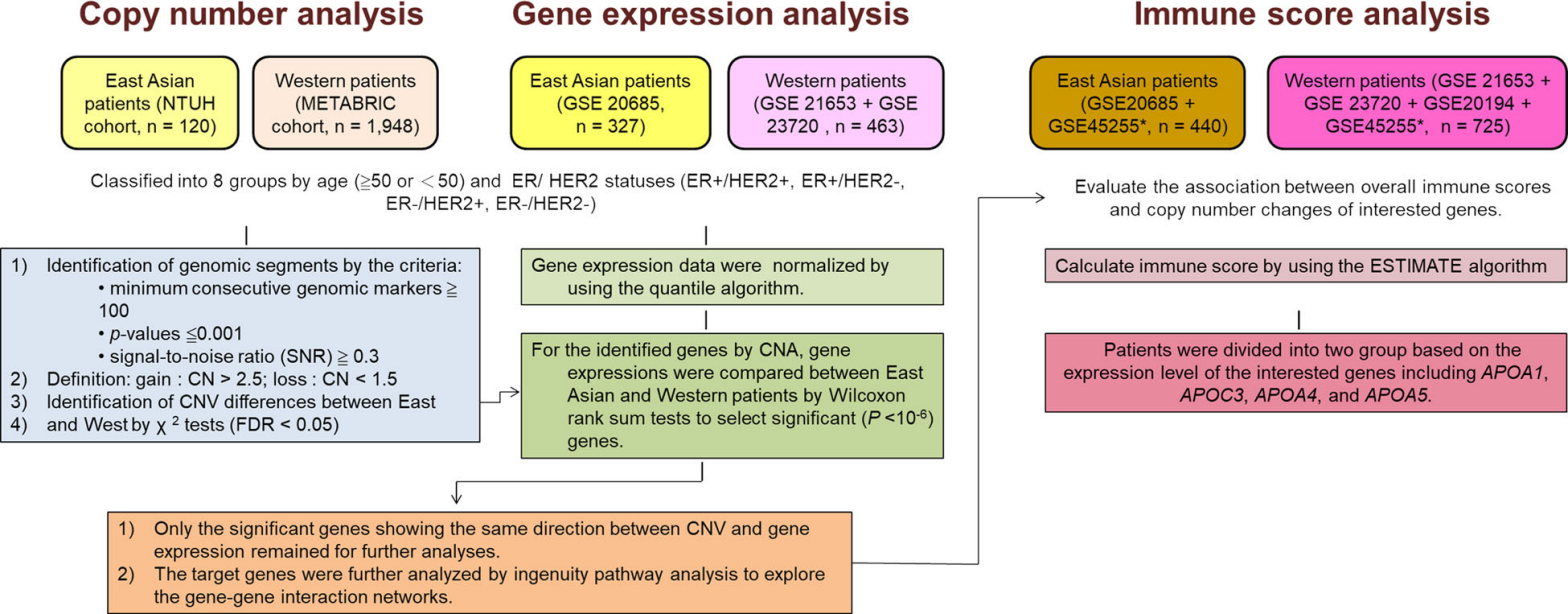
Study flow diagram. The bioinformatics schema and statistical analyses for comparing the genetic differences between the breast tumors of East Asian and Western patients.

Genetic evidence on the distinct biology of ER+/HER2– tumors in young East Asian women remains scant. To fill this gap and to explore how tumor biology differs with respect to race, we compared the copy number variation (CNV) and gene expression (GE) profiles of breast tumors of East Asian and Western women, with stratification by age and ER/HER2 status. The study schema and bioinformatics analyses are illustrated in Fig. 1.

## Results

### CNVs of breast cancers in the discovery cohort

The study cohort included 292 patients with breast cancer from the National Taiwan University Hospital (NTUH). The patients were designated as either the NTUH discovery cohort (*n* = 120) or the validated cohort (*n* = 172) according to the quantity of tumor DNA extracted. To establish the genomics profile of breast cancer in East Asia, we analyzed the genome-wide CNV of 120 breast tumors from the NTUH discovery cohort by using Affymetrix SNP6.0 arrays. To ensure the validity of the CNV being called, we first correlated the copy number of HER2 which was called by the SNP6.0 analysis with the results obtained through routine clinical immunohistochemistry (IHC) and fluorescence in situ hybridization (FISH) assays. A strong correlation of HER2 amplification (defined as a CN ≥4.0) measured by SNP6.0 analysis with overexpression and/or amplification measured by IHC and FISH (*P* < 0.001, Supplementary Table 1) suggested that our CNV analysis was robust. The CNV dataset of the 120 breast tumors in the NTUH discovery cohort was designated as the GSE80526 dataset.^16^

### Copy number−driven genetic networks

To decipher the genetic differences between breast tumors from East Asian and Western patients, we proceeded to compare the CNV profiles of the NTUH discovery cohort with those of the METABRIC (Molecular Taxonomy of Breast Cancer International Consortium) dataset.^17^ The cases were categorized into eight subgroups by age/ER/HER2 status (age: <50 years, ≥50 years; ER+, ER–; and HER2+; HER2–). For each age/ERr5/HER2 subgroup, we aimed to identify the copy number−driven genetic networks that would represent biological functions and signaling pathways. The ER+/HER2+ subtype of both age groups (<50 years and ≥50 years) exhibited no adequate genetic differences for network analysis. The top three networks were identified by using the ingenuity pathway analysis (IPA) based on their connectivity; that is the most connected networks were reported. The top three major networks among the other six age/ER/HER2 subgroups (18 total networks) are presented in Supplementary Fig. 1A–C and summarized in Table 1. First, we used a bird’s-eye view to search across these subgroups for common networks that might serve as the functional pathway nodes. The common nodes were defined as those having three or more occurrences in the top three major networks among the six age/ER/HER2 subgroups, and they included the NFkB (6/18), PI3K (5/18), Akt (3/18), MAPK (6/18), ERK (6/18), IFNα (5/18), Jnk (3/18), Hsp90 (3/18), and Histone h3 (3/18) networks. These networks represented networks that were common among all the breast cancers.

**Table 1:** Three major networks of overlapping differences in GE and CNV in NTUH and METABRIC cohorts.

	Network		
	Top 1	Top 2	Top 3
**Age** <**50 years**			
**ER**+**/HER2−**	NFkB, CCL2, CASP9	PI3K, Akt, JUN, Jnk, IFNα, H2AFX	ERK1, MAPK, AP1, VEGF, APOA1, APOC3
**ER−/HER2**+	MAPK, RARA, ERK1/2, Akt, IFNα, Hsp90, histone h3	MAPK, STAT1, SP1, CDKN1B, RARA, CD40LG, MMP9	TP53, RELA, NR3C1,
**ER−/HER2−**	NFkB, ERK, PI3K, 26 s proteosome	NFkB, BCL2L1, TAC1, Pkc, CALCA	Histone h3, LH, FSH, RNA polymerase II, PDLIM2
**Age** ≥**50 years**			
**ER**+**/HER2−**	NFkB, PI3K, INFα, P38MAPK, Jnk	ERK1/2, Akt, CCDC88A, CYP19A1, focal adhesion kinase, Hsp90, JLN1	MAPK, ERK, Ap1, ER
**ER−/HER2**+	P38MAPK, IFNα, Hsp90, Jnk, RAD51	ERK, ER, histone h3, RNA polymerase II, cyclin A	NFkB, PKc, PI3K, PTK2B, CD3, TCR, ITGB1, PTK2B, BCL10
**ER−/HER2−**	NFkB, PI3K, ITGB3, MYD88	STAT3, STAT1, TNF	CTNNB1, TP53, ESR1, CCND1, TGFBR2, EGFR

### Specific copy number-driven network of *APOA1/C3/A4/A5* in the <50 years/ER+/HER2− subgroup

We subsequently explored the networks unique to each subgroup. For the age <50 years/ER+/HER2− subgroup, which is emerging as the dominant breast cancer population in East Asia^12^, the top first and second networks included nodes that we identified to be common, such as NFkB, PI3K, and Akt (Supplementary Fig. 1A and Table 1). In the third network of this age <50 years/ER+/HER2− subgroup, the networks APOA1 and APOC3 caught our attention. We observed a unique difference in *APOA1, APOC3, APOA4*, and *APOA5* gene alterations. By using the IPA overlay function, the copy number alterations of these four genes were observed to occur in patients aged <50 years (Supplementary Fig. 2A) but not in those aged ≥50 years (Supplementary Fig. 2B).

### Comparison of copy number loss of *APOA1/C3/A4/A5* between NTUH discovery cohort and METABRIC cohort, and validation analysis of NTUH validation cohort in the age <50 years/ER+/HER2− subgroup

**Fig. 2:**
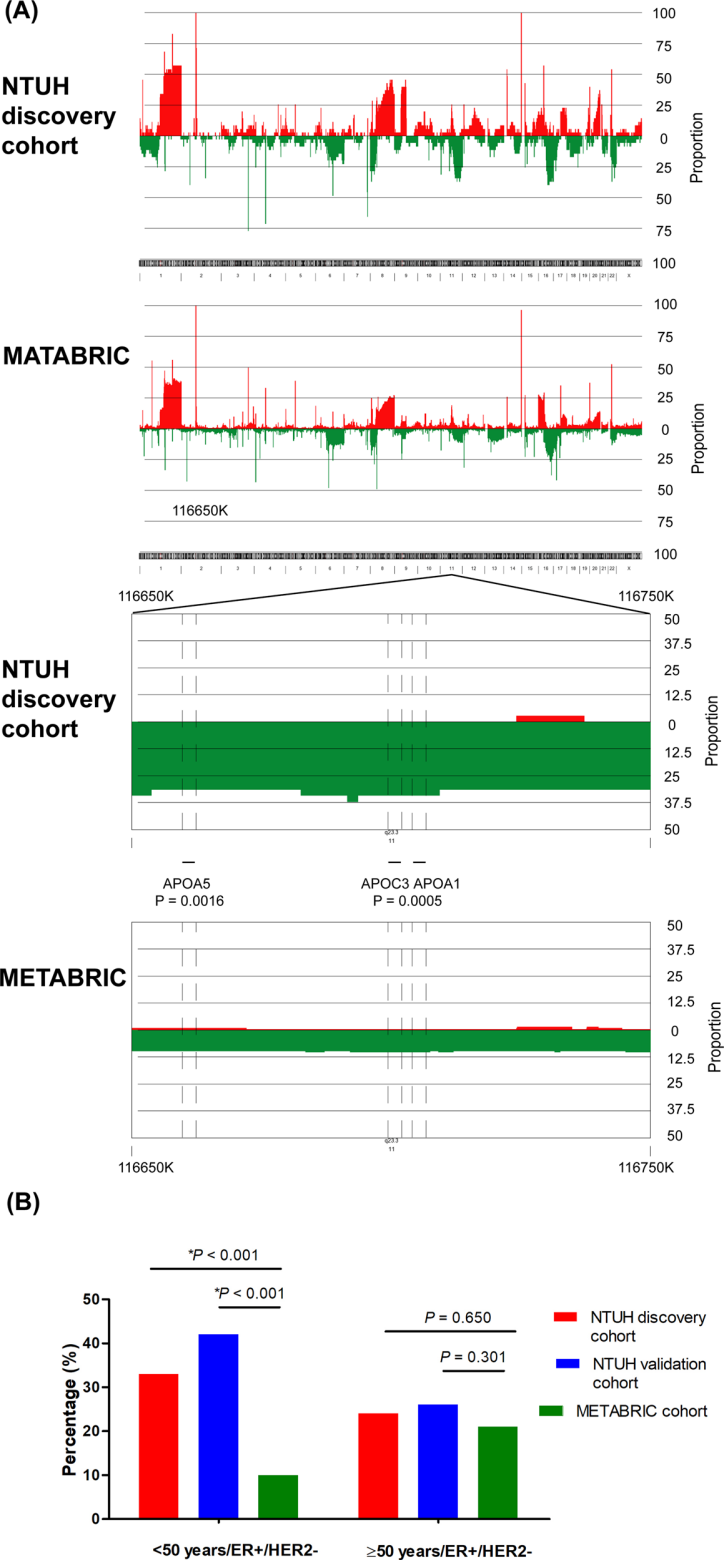
CNV alterations of *APOA1*, *APOC3*, *APOA4*, and *APOA5*. CNV patterns (**A**) and frequencies of *APOA1/C3/A4/A5* or *APOC3/A5* copy number loss (**B**) of breast tumors in age <50 years/ER+/HER2- subgroup of East Asian (NTUH discovery cohort and/or NTUH validation cohort) and Western (METABRIC cohort) patients.

*APOA1/C3/A4/A5* is a gene cluster located on chromosome 11q23 and it is crucial to the modulation of lipid metabolism.^18^ The CNV frequencies along the 23 chromosomal regions corresponding to the *APOA1/C3/A4/A5* gene cluster in the age <50 years/ER+/HER2− in NTUH discovery cohort and METABRIC dataset are illustrated in Fig. 2A, and those in age ≥50 years/ER+/HER2− subgroup are illustrated in supplementary Fig 3. In the age <50 years/ER+/HER2− subgroup, the frequencies of *APOA1/C3/A4/A5* loss were significantly higher in the tumors of the NTUH discovery cohort than in those of the METABRIC cohort (33 vs. 10%, *P* < 0.001, Fig. 2B).

To confirm the copy number loss of *APOA1/C3/A4/A5*, we selectively validated the copy number of *APOC3* and *APOA5* by using the TaqMan copy number assay in 33 cases from the NTUH discovery cohort. *APOC3* and *APOA5* deletions that were defined by using the TaqMan copy number assay were well correlated with those defined by using the Affymetrix SNP6.0 arrays (94% concordance rate for Hs07444493_cn of *APOC3*, presented in Supplementary Fig. 4A; 76% concordance rate for Hs02219432_cn of *APOA5*, and 85% concordance rate for Hs02106826_cn of *APOA5*, presented in Supplementary Fig. 4B)^19^. We further evaluated the CNVs of *APOC3* and *APOA5* in 172 cases from the NTUH validation cohort by using the TaqMan copy number assay. Copy number losses of both *APOC3* and *APOA5* were identified in 50 cases (29%).

### Clinicopathological significance of *APOA1/C3/A4/A5* or *APOC3/A5* loss

The associations between the copy number loss of *APOA1/C3/A4/A5* or *APOC3/A5* and clinicopathological features are presented in Table 2. The frequencies of *APOA1/C3/A4/A5* or *APOC3/A5* copy number loss were higher in the age <50 years subgroups of the East Asian cohorts than in counterpart subgroups of the Western cohort (NTUH discovery cohort: 32%; NTUH validation cohort: 37%; METABRIC cohort: 7%, both *P* values < 0.001), but the differences were nonsignificant in the age ≥50 years subgroups (NTUH discovery cohort: 23%; NTUH validation cohort: 24%; METABRIC cohort: 19%). The frequency of *APOA1/C3/A4/A5* or *APOC3/A5* copy number loss was significantly and marginally significantly higher in the ER + tumors than in the ER − tumors in METABRIC and NTUH discovery cohorts, respectively. The frequencies of *APOA1/C3/A4/A5* or *APOC3/A5* copy number loss in age <50 years/ER+/HER2− subgroup were significantly higher in the NTUH discovery or NTUH validation cohort than in the METABRIC cohort, but the differences were nonsignificant in the age ≥50 years/ER+/HER2− subgroups (Fig. 2B).

**Table 2:** Associations of *APOA1/APOC3/APOA4/APOA5* or *APOC3/APOA5* copy number loss with clinicopathological features.

	NTUH discovery cohort^a^			NTUH validation cohort^†^			METABRIC cohort^‡^		
Characteristics	*APOA1/C3/A4/A5*, No. (%)			*APOC3/A5*, No. (%)			*APOA1/C3/A4/A5*, No. (%)		
	loss (*n* = 33)	normal (*n* = 86)	*P*	loss (*n* = 50)	normal (*n* = 120)	*P*	loss (*n* = 324)	normal (*n* = 1650)	*P*
Age (years)			0.299			0.072			<0.001
<50 years	20 (32)	43 (68)		26 (37)	45 (63)		29 (7)	393 (93)	
≥50 years	13 (23)	43 (77)		24 (24)	75 (76)		295 (19)	1257 (81)	
Histologic grade			0.838			0.834			0.460
1	7 (30)	16 (70)		5 (23)	17 (77)		25 (15)	145 (85)	
2	12 (29)	30 (71)		21 (29)	51 (71)		138 (18)	632 (82)	
3	13 (25)	40 (76)		19 (27)	51 (73)		152 (16)	792 (84)	
unclassified	1	0		5	1		9	81	
Stage			0.341			0.612			0.182
I	4 (25)	12 (75)		9 (25)	27 (75)		72 (14)	441 (86)	
II	21 (33)	43 (67)		24 (27)	64 (73)		146 (17)	698 (83)	
III	5 (16)	26 (84)		12 (36)	21 (64)		20 (12)	146 (88)	
IV	3 (38)	5 (64)		5 (38)	8 (62)		1 (10)	9 (90)	
unknown	0	0					85	365	
ER status			0.094			0.324			<0.001
Negative	8 (19)	35 (81)		14 (24)	43 (76)		20 (5)	410 (95)	
Positive	25 (33)	51 (67)		36 (32)	77 (68)		300 (20)	1200 (80)	
unknown	0	0		0	0		4	40	
PR status			0.393			0.890			0.110
Negative	14 (24)	44 (76)		24 (29)	59 (71)		140 (15)	793 (85)	
Positive	19 (31)	42 (69)		26 (30)	61 (70)		184 (18)	857 (82)	
HER2 status			0.984			0.349			0.721
Negative	25 (28)	65 (72)		34 (27)	90 (73)		250 (16)	1288 (84)	
Positive	8 (28)	21 (72)		16 (35)	30 (65)		74 (17)	362 (83)	

^†^Two cases with a copy number gain for APOC3/A5 were excluded.

^‡^Eighteen cases with a copy number gain for APOA1/C3/A4/A5 were excluded.

^a^One case with a copy number gain for APOA1/C3/A4/A5 was excluded.

*APOA1/C3/A4/A5* and *APOC3/A5* copy number losses were not associated with disease-free survival in patients with stage I–III breast cancer in NTUH discovery (Supplementary Fig. 5A) and validation cohorts (Supplementary Fig. 5B), respectively. In the METABRIC cohort, *APOA1/C3/A4/A5* copy number loss was significantly associated with shorter breast cancer-specific survival in both the univariate analysis (hazard ratio [HR] = 1.33, *P* = 0.011) (Supplementary Fig. 5C) and the multivariate analysis (HR = 1.44, *P* = .007) (Table 3).

**Table 3:** Association of breast cancer outcome and *APOA1/C3/A4/A5* or *APOC3/A5* loss: the univariate analyses in the three cohorts and a multivariate Cox’s proportional hazards model in METABRIC cohort.

	HR	95% CI	*p* value
**Univariate analysis**			
NTUH discovery cohort^a^			
*APOA1/C3/A4/A5* loss versus normal	0.78	0.25–2.34	0.634
NTUH validation cohort^a^			
*APOC3/A5* loss versus normal	1.24	0.44–3.49	0.683
METABRIC cohort^b^			
*APOA1/C3/A4/A5* loss versus normal	1.33	1.07–1.65	0.011
**Multivariate analysis of METABRIC cohort**^b^			
*APOA1/C3/A4/A5*			
loss versus normal	1.44	1.11–1.87	0.007
Stage			
II versus I	2.20	1.69–2.88	<0.001
III versus I	5.02	3.66–6.88	<0.001
IV versus I	15.13	5.47–41.88	<0.001
Histological grade			
II versus I	1.50	0.86–2.61	0.154
III versus I	1.87	1.08–3.25	0.026
ER status			
positive versus negative	0.67	0.52–0.85	0.001
HER2 overexpression			
yes versus no	1.41	1.12–1.77	0.003

^a^Patients with stage I–III breast cancer were included, and disease-free survival was used as the survival endpoint.

^b^Patients with stage I–IV breast cancer were included, and breast cancer-specific survival was used as the survival endpoint.

### Comparison of GEs of *APOA1/C3/A4/A5* of breast tumors between Asians and Caucasians in the age <50 years/ER+/HER2− subgroup

**Fig. 3:**
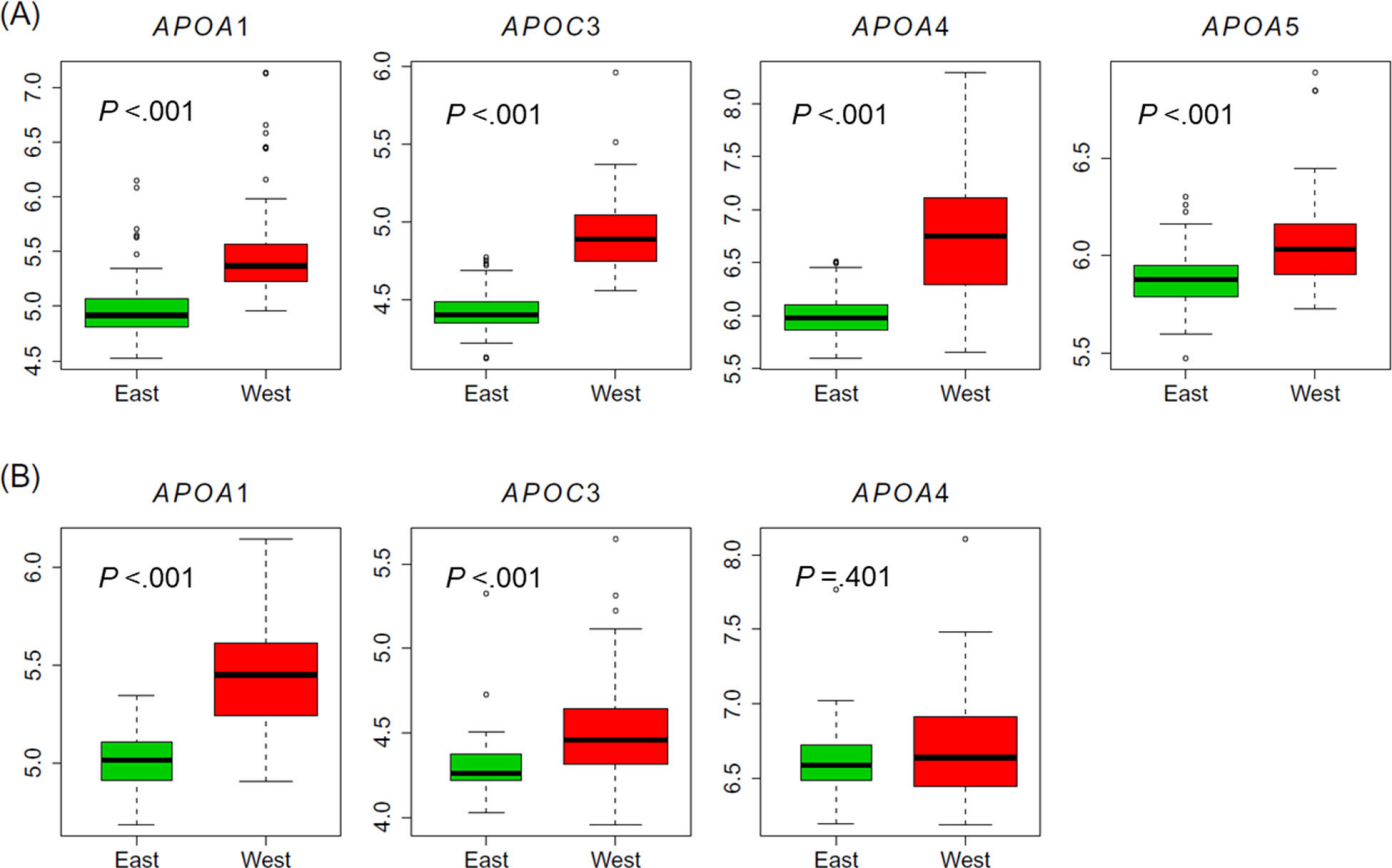
GEs of *APOA1*, *APOC3*, *APOA4*, and *APOA5*. Comparisons of *APOA1*, *APOC3*, *APOA4*, and *APOA5* gene expressions the ER+/HER2- tumors between East Asian (East) and Western patients (West) aged <50 years by analyzing GSE20685, GSE21653, and GSE23720 datasets (**A**), and by GSE2019 and GSE45255 datasets (**B**).

To compare the GEs of the *APOA1/C3/A4/A5* in breast tumors of the age <50 years/ER+/HER2− subgroup between Asians and Caucasians, we retrieved the raw data from the GSE20194^20^ and GSE45255^21^ datasets, which included tumors from multiple races. In the age <50 years/ER+/HER2− subgroup, these two datasets included data on 30 Asian and 64 Caucasian patients. The GEs of these two datasets were profiled using an Affymetrix Human Genome U133 A Array, with probes for APOA1, APOC3, and APOA4, but not APOA5. In agreement with the high prevalence of *APOA1/C3/A4/A5* copy number loss, the GEs of the four genes were determined to be significantly lower in the ER+/HER2− tumors of East Asian patients aged <50 years relative to the tumors of their Western counterparts based on an analysis of the GSE20685^22^, GSE21653^23^, and GSE23720^24^ datasets (Fig. 3A). In the GSE20194^20^ and GSE45255^21^ datasets, *APOA1* and *APOC3* expressions were significantly lower in the ER+/HER2− tumors of East Asian patients aged <50 years than in those of Western patients. *APOA4* expression did not differ significantly between the two groups (Fig. 3B).

### Inverse association between *APOA1*, *APOC3*, and *APOA5* expressions and Estimation of STromal and Immune cells in MAlignant Tumors (ESTIMATE) immune scores

Several studies have documented the association between decreased APOA1/C3/A4/A5 levels and chronic inflammation.^18,25–27^ We conducted a discovery analysis of five GE profiles, GSE20685^22^, GSE21653^23^, GSE23720^24^, GSE20194^20^, and GSE45255^21^, to examine the association between *APOA1/C3/A4/A5* expression and ESTIMATE immune score. The ESTIMATE algorithm was used to determine the immune score which inferred the abundance of tumor-infiltrating immune cells.^28^ In the East Asian subgroup, expressions of *APOA1, APOC3, and APOA5* were negatively associated with ESTIMATE immune scores. In the Western subgroup, the associations between *APOA1* and *APOC3* expressions and ESTIMATE immune score were consistently significant, but the association between *APOA5* and ESTIMATE immune score was only marginally significant. *APOA4* expression was not associated with ESTIMATE immune score in the East Asian subgroup, but was positively associated with ESTIMATE immune score in the Western subgroup (Table 4).

**Table 4:** Associations of expressions of *APOA1, APOC3, APOA4*, *and APOA5* with ESTIMATE immune score in breast tumors of East Asian and Western patients.

	East Asian		Western	
	Immune score^a^	*P*	Immune score^a^	*P*
***APOA1***		<0.001		0.019
High	1328.5 (34.5)		1297.2 (43.4)	
Low	1507.1 (33.8)		1447.9 (46.9)	
***APOC3***		0.006		<0.001
High	1351.5 (35.1)		1249.5 (44.0)	
Low	1484.1 (33.4)		1495.5 (45.8)	
***APOA4***		0.797		<0.001
High	1424.1 (35.1)		1472.3 (47.6)	
Low	1411.6 (33.6)		1273.8 (42.4)	
***APOA5***^b^		0.002		0.057
High	1290.0 (42.5)		1187.9 (53.0)	
Low	1479.2 (42.3)		1333.7 (54.8)	

^a^Mean (Standard error).

^b^Total 327 Asian and 463 Western in APOA5 analysis.

## Discussion

In the present study, we report the presence of somatic genetic differences between East Asian and Western patients with breast cancer by age group and ER/HER2 subtype. For the age <50 years/ER+/HER2– subgroup, we identified *APOA1/C3/A4/A5* copy number loss as a unique gene alteration in the tumors of East Asian patients. The frequency of *APOA1/C3/A4/A5* copy number loss was higher and the expressions of these four genes were lower in the tumors of the East Asian patients than in those of Western patients. The lower levels of *APOA1*, *APOC*3, and *APOA5* expression were associated with higher ESTIMATE immune scores, which indicated an abundance of tumor-infiltrating immune cells. *APOA1/C3/A4/A5* copy number loss was associated with poor survival in Western patients, but in the East Asian patients.

The strength of this study is that we systemically compared the genetic differences between the tumors of East Asian and Western patients by applying an integrated whole-genome approach to our analysis of the CNV and GE; this application can enhance the identification of the relevant functional genes and reveal the biological mechanisms.^29–31^ To avoid the bias caused by comparison across datasets, we selected datasets that used the same array platforms. The integrated analysis revealed that the common nodes among the six age/ER/HER2 subgroups included *NFkB*, *PI3K*, *Akt*, *MAPK*, *ERK*, *IFNα, and Jnk* networks. Among the networks, the expressions of some key genes such as *MAPK* and *ERK* were lower in the tumors of East Asian patients. This finding suggests the presence of relatively indolent tumor biology among East Asian patients, and it is consistent with studies which have demonstrated that Chinese and Japanese women develop less aggressive breast tumors than their Western counterpart.^13,32,33^

In the age <50 years/ER+/HER2− subgroup, we observed decreased copy numbers and GEs for the *APOA1/C3/A4/A5* genes in tumors of East Asian patients. The *APOA1/C3/A4/A5* gene cluster transcribes the apolipoproteins A1, C3, A4, and A5, which regulate high-density lipoprotein assembly and lipoprotein lipase activity. These apolipoproteins shuttle excess cholesterol and triglycerides from peripheral tissues to the liver for excretion.^18,34^ Decreased levels of these apolipoproteins can result in lipid accumulation and chronic inflammation within the arterial wall, and eventually lead to atherosclerosis and cardiovascular disease. Polymorphisms of *APOA1/C3/A4/A5* have been demonstrated to be associated with metabolic syndrome, dyslipidemia, and cardiovascular diseases.^18,34,35^ Among them, the APOA1 protein has been extensively studied and has been demonstrated to protect against tumor development or progression in several cancers.^26,36,37^ Serum APOA1 level is also negatively associated with higher breast cancer risk^38^ and recurrence^39^.

Although the exact mechanism of APOA1 protein antitumor activity is unclear, its immunomodulatory and anti-inflammatory effects on the tumor microenvironment exert the protective activity in mouse tumor models.^26,40,41^ APOA1 protein can reduce the recruitment of myeloid-derived suppressor cells, moderate antiangiogenesis, and increase the number of CD8 + T cells.^26,36,40,42,43^ The present study revealed that *APOA1* expression is negatively associated with ESTIMATE immune score in breast tumors among both East Asian and Western patients. This finding supports studies that have demonstrated that APOA1 protein can modulate the tumor microenvironment. The associations are similar for *APOC3* and *APOA5*. By contrast, the association of *APOA4* with ESTIMATE immune score differed from those of *APOA1*, *APOC3*, and *APOA5* with ESTIMATE immune score; however, the reason underlying this finding remains unclear. Notably, a prior study observed APOA1 and APOA4 had different functional structural characteristics in their lipid-free states and that APOA4 exhibited less anti-atherogenic activity.^44^

A meta-analysis of prospective cohort studies revealed that elevated serum cholesterol levels are associated with breast cancer risk in Asians but not in Caucasians.^45^ The different effects of cholesterol levels on breast cancer risks and the prevalence of *APOA1/C3/A4/A5* copy number loss in the tumors of East Asian patients suggests that the dysregulation of lipid metabolism plays different roles in breast cancer between Western and East Asian women. We hypothesize that ethnic differences in lipid metabolism or apolipoprotein gene polymorphisms may explain this discrepancy. For example, an immigration study revealed that *APOA1* polymorphisms and patterns are common among people with Asian ethnicity, but the patterns and polymorphisms differ from those reported in European Caucasians.^46^ To explain why the racial difference of *APOA1/C3/A4/A5* copy number loss manifested in young patients but not in old patients, we hypothesize that the increasing lipid intake in young East Asian women with gene polymorphisms of impaired lipid metabolism contributes to a higher incidence rate of breast cancer with *APOA1/C3/A4/A5* copy number loss.

The prognostic value of *APOA1/C3/A4/A5* copy number loss differed between East Asian and Western patients. Our study demonstrated that *APOA1/C3/A4/A5* copy number loss was significantly associated with lower survival in patients with breast cancer, although the association was only significant for Western patients. We further examined the prognostic values of the mRNA expressions of *APOA1, APOC3, APOA4*, and *APOA5* by using two online survival analysis software packages that were established through analyses of predominantly Western patients with breast cancer. Among expressions of these four genes, a decreased expression of *APOA1* was associated with shorter survival according to results from Kaplan–Meier plotter software (http://kmplot.com/analysis/^47^; relapse-free survival, HR = 1.15, *P* = 0.011) and The Human Protein Atlas (https://www.proteinatlas.org/pathology^48^; 5-year overall survival rate 78 vs. 85%, *P* = 0.009). *APOA1/C3/A4/A5* copy number loss may result in the recruitment of myeloid-derived suppressor cells or the induction of angiogenesis, which would lead to poor patient outcomes. It remains unclear why *APOA1/C3/A4/A5* copy number loss was not associated with patient outcome in East Asian patients. Prior studies and our recent study have reported the presence of greater immune activity in the breast tumor microenvironment of Asian patients than that of Caucasian patients.^49–53^ In our recent study, a high ESTIMATE immune score was associated with shorter disease-free survival in the luminal A subtype of the Western patients, but a trend toward longer overall survival in the luminal B subtype of Asian patients.^53^ *APOA1/C3/A4/A5* copy number loss may have an attenuated influence in the tumor microenvironment of Asian patients.

Finally, this study has some limitations. First, the comparison of genetic alterations of breast tumors between Asian and Western patients was indirect. To reduce the bias of indirect comparisons, we applied an integrated approach to our whole-genome analysis of the CNV and GE, and we validated the differences in both of the GEs and copy number assays. Second, we divided patients into NTUH discovery cohort and validation cohort according to the quantity of extracted tumor DNA. Although the NTUH validation cohort was not an ideally independent cohort, the similarities in clinicopathological features between NTUH discovery and validation cohort (presented in Table 2) suggest that the various amounts of tumor DNAs were due to unequal sample collections or variation in tumor cellularity. Third, although the majority of patients included in the Western database were Caucasian, a small percentage of patients of Asian descent included in the Western databases could have been misclassified as Western patients. Similarly, a certain percentage of patients of African descent were also included in the Western databases. Fourth, the differences in tumor genetic alterations between East Asian and Western patients can be attributed to genetic or environmental factors. Without another group of patients of Asian descent living in Western countries, our study cannot differentiate between genetic and environmental factors.

In summary, we demonstrated the somatic genetic differences in breast cancer between East Asian and Western patients by integrating analyses of CNV and GE. The reduced activation of the *NFkB*, *PI3K*, *Akt*, *MAPK*, *ERK*, *IFNα*, and *Jnk* networks in breast cancer of East Asian patients suggested the presence of relatively indolent tumor biology. We identified some unique differences in the age ≤50 years/ER+/HER2− tumors subgroup, and we selectively validated and verified that the copy number loss of *APOA1/C3/A4/A5* constituted a unique genetic alteration. Mechanistically, our discovery analysis indicated that expressions of *APOA1*, *APOC3*, and *APOA5* are inversely associated with tumor-infiltrating immune cells, and we proposed that they exert an immunomodulatory effect on the tumor microenvironment. *APOA1/C3/A4/A5* copy number loss was significantly associated with poor survival in Western patients but not in East Asian patients. Further research on the biology and changes in *APOA1/C3/A4/A5* genes and other gene alterations in breast cancer cells is warranted.

## Methods

### Schema and bioinformatics analyses

As illustrated in Fig. 1, we compared the CNV and GE profiles of breast tumors between women from East Asian and Western patients, with stratifications by age and ER/HER2 status. Overlapping genes identified in the analysis of CNV and GE profiles were selected to construct networks representing the CNV–related differentially expressed gene signaling in the breast tumors between East Asian and Western patients. In the present study, we focused on the age <50 years/ER+/HER2– subgroup, which is a subpopulation in East Asia among which breast cancer is rapidly increasing.

### Breast cancer cohorts at NTUH

Freshly frozen primary tumors and matched blood samples were collected from 292 patients with breast cancer diagnosed between April 2009 and July 2011 at NTUH, Taiwan. Written informed consent was obtained, and the study’s protocol was approved by the ethics committee of NTUH (200902014 R). The clinicopathological information of these patients is summarized in Supplementary Table 2. Genomic DNA was extracted from the blood and tumor specimens by using commercial kits per the manufacturer’s protocol (Qiagen DNeasy Kit). In particular, ≥500 and <500 ng of tumor DNAs were collected from 120 and 172 tumors, respectively, which comprised the NTUH discovery cohort and NTUH validation cohort.

### Genome-wide copy number profiling

The matched genomic and tumor DNAs from the 120 discovery cases were hybridized by using Affymetrix SNP6.0 arrays (Affymetrix, Santa Clara, CA, USA) per the manufacturer’s instructions. The experiment was conducted at the National University of Singapore. The CNV data were obtained from METABRIC dataset^17^, the largest publicly available breast cancer dataset that Affymetrix SNP6.0 array was used. After we excluded 44 patients who had no documented ER or HER2 status, CNVs from 1948 patients were analyzed.

### Bioinformatics analysis of CNV

The raw.cel CNV files were imported into Partek Genomic Suite 6.5 (Partek, St Louis, Missouri, USA), and the genomic segments were defined according to the following criteria: minimum consecutive genomic markers of 100, *P* ≤ 0.001, and signal-to-noise ratio ≥0.3.^54^ A segment was defined as a copy number variant region (CNVR) if its copy number was ≥2.5 (amplification) or ≤1.5 (deletion).^29^ A chi-square test (false discovery rate <0.05) and Fisher’s exact test (*P* < 0.05) were subsequently performed to identify CNVRs that exhibited significant differences between the tumors of the East Asian and Western patients. The gene annotation file from the reference sequence (refseq) database was used to identify the gene symbols of the significant CNVRs based on the refseq transcript file released on April 29, 2014, in the Partek Genomic Suite.

### Breast cancer GE profiles

To compare the GE profiles in breast tumors between East Asian and Western patients, we obtained the raw data of the GSE20685 dataset^22^, which included 327 breast tumors from Taiwanese patients, and the GSE21653^23^ and GSE23720^24^ datasets, which included 423 breast tumors from French patients. The GE profiling of these three datasets was conducted using the Affymetrix Human Genome U133 Plus 2.0 Array. The raw.cel files of the three datasets were retrieved from the Gene Expression Omnibus. We performed a quantile normalization algorithm analysis on the GSE20685 dataset^22^ to obtain the reference baseline because it had the largest sample size. We subsequently adjusted the GSE21653^23^ and GSE23720^24^ datasets to the reference baseline by using a quantile normalization algorithm. To avoid bias caused by the examination or the positivity cutoff of the ER/HER2 status, the ER and HER2 statuses among these three datasets were determined in accordance with the GE intensity which was measured using a microarray, per a method in a previous study.^55^ The numbers of included patients, stratified by age and ER/HER2 status, are presented in Supplementary Table 3.

### Genetic networks by overlapping differences in CNV and GE

The identified genes in our CNV analysis were further examined by using the Wilcoxon rank-sum test to investigate whether tumor GE levels significantly (*P* < 10^−6^) differed between East Asian (GSE20685^22^) and Western (GSE21653^23^ and GSE23720^24^) patients. The genes that met the stringent criteria in GE comparisons and exhibited the same direction in CNV and GE analyses were employed to define the copy number-driven genetic networks using IPA.^54,56^

### Validation of *APOA1/C3/A4/A5* CNV and GE

To validate the CNVs of *APOA1/C3/A4/A5*, we selectively examined the copy number of *APOC3* and *APOA5* through real-time qualitative polymerase chain reaction experiments (qPCR) by using the ABI 7900HT system (Applied Biosystems, Foster City, CA, USA) in NTUH discovery and validated cohorts. We used the TaqMan assay in the following manner: Hs07444493_cn was used for *APOC3* and Hs02219432_cn and Hs02106826_cn were used for *APOA5*. A human ribonuclease P TaqMan copy number reference assay was used as an endogenous control for two copies in the human genome. The thermal cycling conditions were set at 95 °C for 10 min, 40 cycles of 95 °C for 15 s, and 60 °C for 1 min. The exported qPCR result files were imported into CopyCaller Software 2.0 (Applied Biosystems), and the copy numbers of the targets were quantified.

To validate the GEs of the *APOA1/C3/A4/A5*, we retrieved the raw data from the GSE20194^20^ and GSE45255^21^ datasets which included tumors from patients of multiple races. The GEs of these two datasets were profiled using an Affymetrix Human Genome U133A Array, with probes for *APOA1, APOC3*, and *APOA4*, but not *APOA5*.

### Analysis of association between *APOA1/C3/A4/A5* expressions and ESTIMATE immune scores

The ESTIMATE algorithm was used to estimate the immune score which inferred the abundance of infiltrating immune cells.^20^ We analyzed five GE profiles: GSE20685^22^, GSE21653^23^, GSE23720^24^, GSE20194^20^, and GSE45255^21^. Per a procedure described in our prior study^53^, the GE profiles were normalized using the quantile normalization algorithm, and the ESTIMATE algorithm was executed to determine the overall immune scores of each sample in each dataset.

### Statistical analysis

The associations between the clinicopathological variables and the *APOA1/C3/A4/A5* or the *APOC3/A5* copy number statuses were examined using chi-square tests. The survival outcomes were estimated using the Kaplan–Meier method. A multivariate analysis was conducted by fitting Cox proportional hazards model to estimate the adjusted effects of predictors (including stage, histological grade, ER status, HER2 status, and *APOA1/C3/A4/A5* copy number status) on the breast cancer-specific mortality rate. Statistical significance was indicated by a two-sided *P* value ≤ 0.05.

### Reporting Summary

Further information on research design is available in the Nature Research Reporting Summary linked to this article.

## Supplementary information

Supplementary Information

Reporting Summary

## Data Availability

The data generated and analysed during this study are described in the following data record: 10.6084/m9.figshare.14696499^19^. The CNV dataset of the 120 breast tumors in the NTUH discovery cohort has been deposited in the Gene Expression Omnibus (GEO) under accession https://identifiers.org/geo:GSE80526^16^. The Metabric dataset is available in the European Nucleotide Archive under accession https://identifiers.org/ena.embl:EGAD00010000164^17^. The breast cancer gene expression datasets used for comparison between East Asian and Western women are available in GEO under accessions: https://identifiers.org/geo:GSE20685^22^, https://identifiers.org/geo:GSE21653^23^, https://identifiers.org/geo:GSE23720^24^, https://identifiers.org/geo:GSE20194^20^, and https://identifiers.org/geo:GSE45255^21^. Clinical data in the files “NTUH discovery cohort patient data.xlsx” and “NTUH validation cohort patient data.xlsx” are not publicly available as they contain information that could compromise research participant privacy and informed consent to share participant-level data was not obtained prior to or during data collection. Requests for these data should be directed to Dr. Lin CH with data requests. The file “TaqMan Copy Number Assay data.xlsx” is openly available with this data record^19^.

## Code availability

Details on the code used in this study are available upon request.
