# Supplementary material for: High prevalence of APOA1/C3/A4/A5 alterations in luminal breast cancers among young women in East Asia
Source: NPJ Breast Cancer. 2021 Jul 5;7:88. doi: 10.1038/s41523-021-00299-5 (PMC8257799; doi:10.1038/s41523-021-00299-5)

Supplementary

Supplementary Table 1 Comparison of HER2 status between CNV and immunohistochemistry and fluorescence in situ hybridization in NTUH exploratory cohort

	HER2 by SNP array		
	positive	negative	***P* value**
**HER2 by IHC and FISH**			<0.001
positive	26	3	
negative	5	86	

Supplementary Table 2 The clinicopathological information of breast cancer patients in NTUH discovery cohort and NTUH validation cohort

	**N (%)**	
Characteristics	NTUH discovery cohort (n = 120)	NTUH validation cohort (n = 172)
Age		
Range (years)	24 – 79	30- 90
<50 years	63 (53)	72 (42)
≥50 year	57 (48)	100 (58)
Histology type		
Ductal	115 (96)	168 (98)
Lobular	1 (1)	1 (1)
Others	4 (3)	3 (2)
Histologic grade		
1	23 (19)	22 (13)
2	43 (36)	72 (43)
3	53 (45)	72 (43)
Unclassified	1	6
Stage		
I	16 (13)	37 (22)
II	65 (54)	89 (52)
III	31 (26)	33 (19)
IV	8 (7)	13 (8)
ER status		
Negative	44 (37)	57 (33)
Positive	76 (63)	115 (67)
PR status		
Negative	59 (49)	84 (49)
Positive	61 (51)	88 (51)
HER2 status		
Negative	90 (75)	124 (72)
Positive	30 (25)	48 (28)

Supplementary Table 3 Patient number of the cohorts and datasets used in the present study

	Copy number analysis			Gene expression analysis		
	NTUH discovery cohort (East)	NTUH validation cohort (East)	METABRIC (West)	GSE20685 (East)	GSE21653 & GSE23720 (West)	GSE20194 & GSE45255 (East and West)
**Age <50 y**						
ER+/ HER2+	7	10	39	10	17	16
ER+/HER2-	35	43	193	109	81	94
ER-/HER2+	8	8	69	34	22	12
ER-/HER2-	13	11	117	50	57	30
**Age ≥50 y**						
ER+/ HER2+	8	9	211	12	27	21
ER+/HER2-	26	53	1065	73	145	148
ER-/HER2+	9	21	81	19	31	19
ER-/HER2-	14	17	173	20	83	35

Supplementary Fig. 1 The top three networks of CNV-driven GE differences of breast tumors between East Asian patients and Western patients in ER+/HER2- (A), ER-/ HER2+ (B), and ER-/ HER2- (C)

(A)

**<50 years/ER+/HER2-**

First network

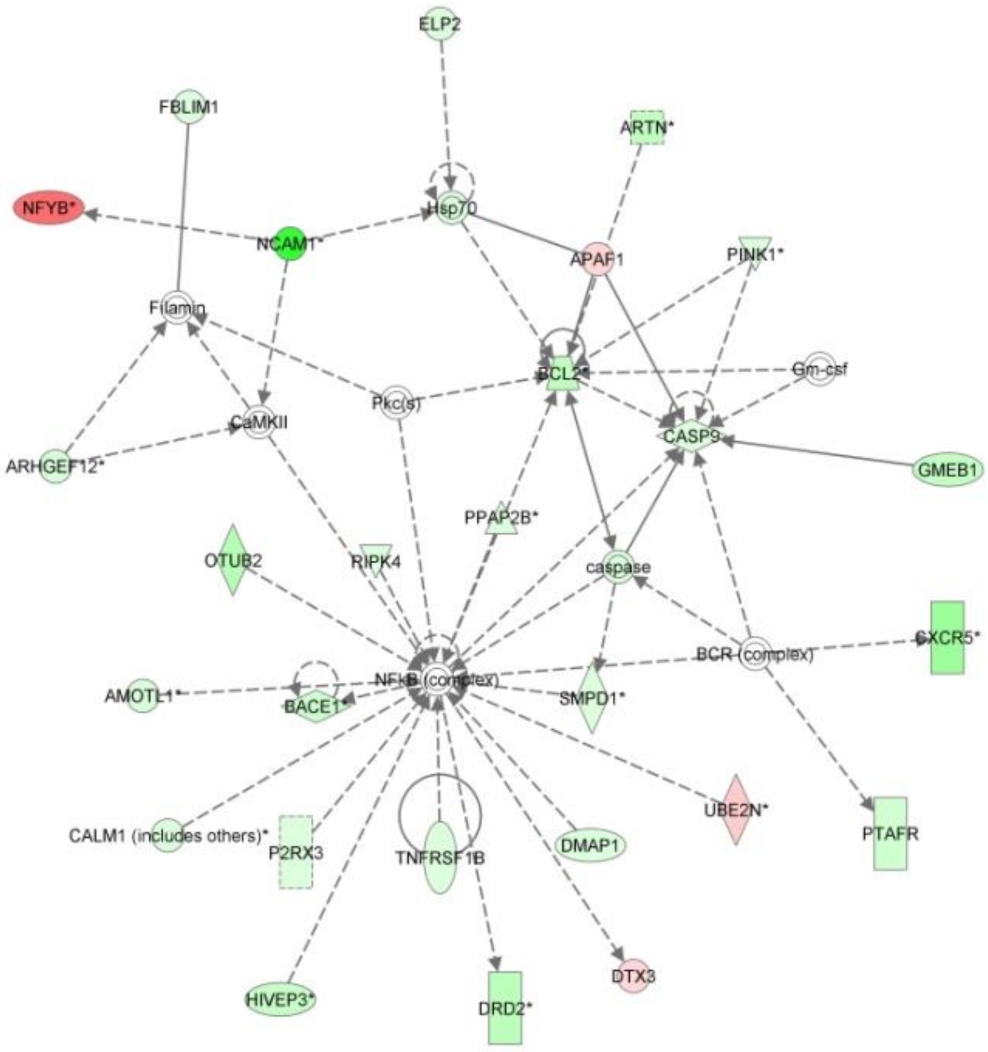

Second network

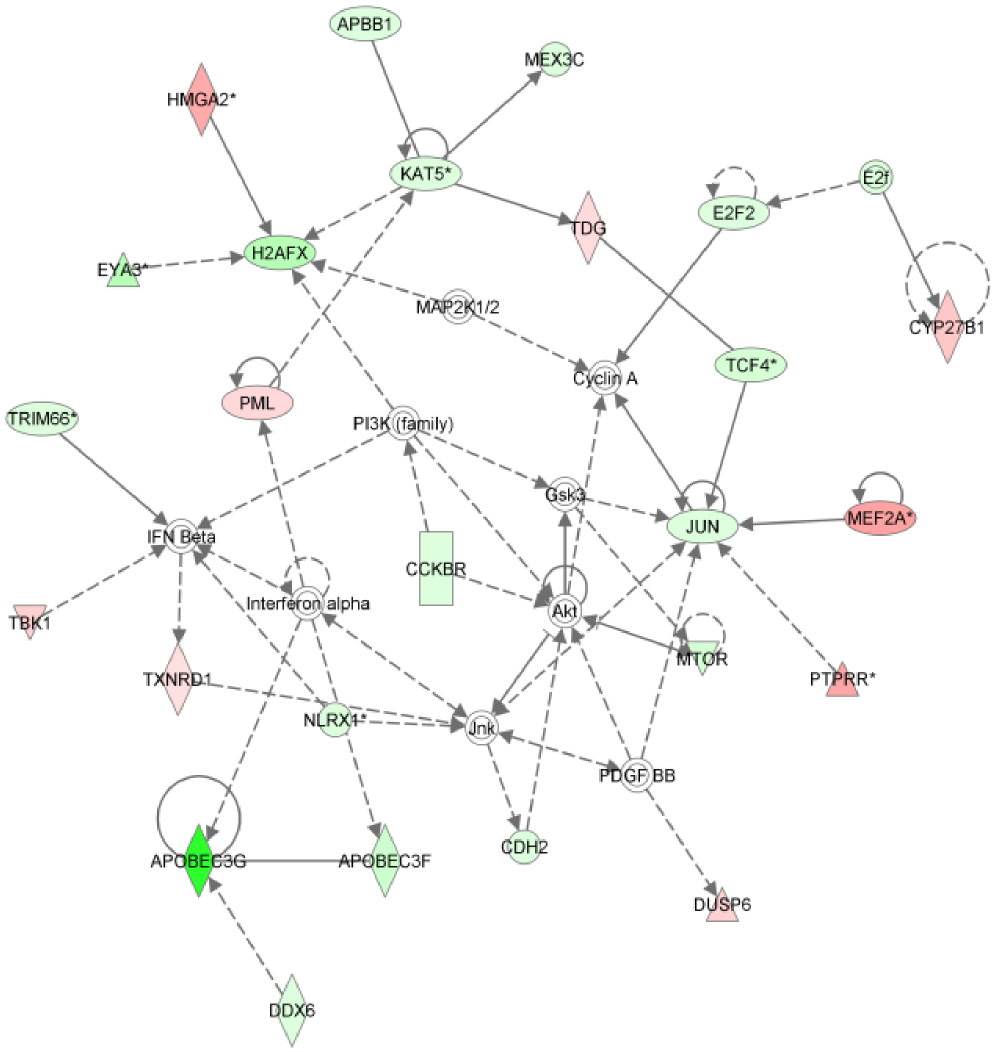

Third network

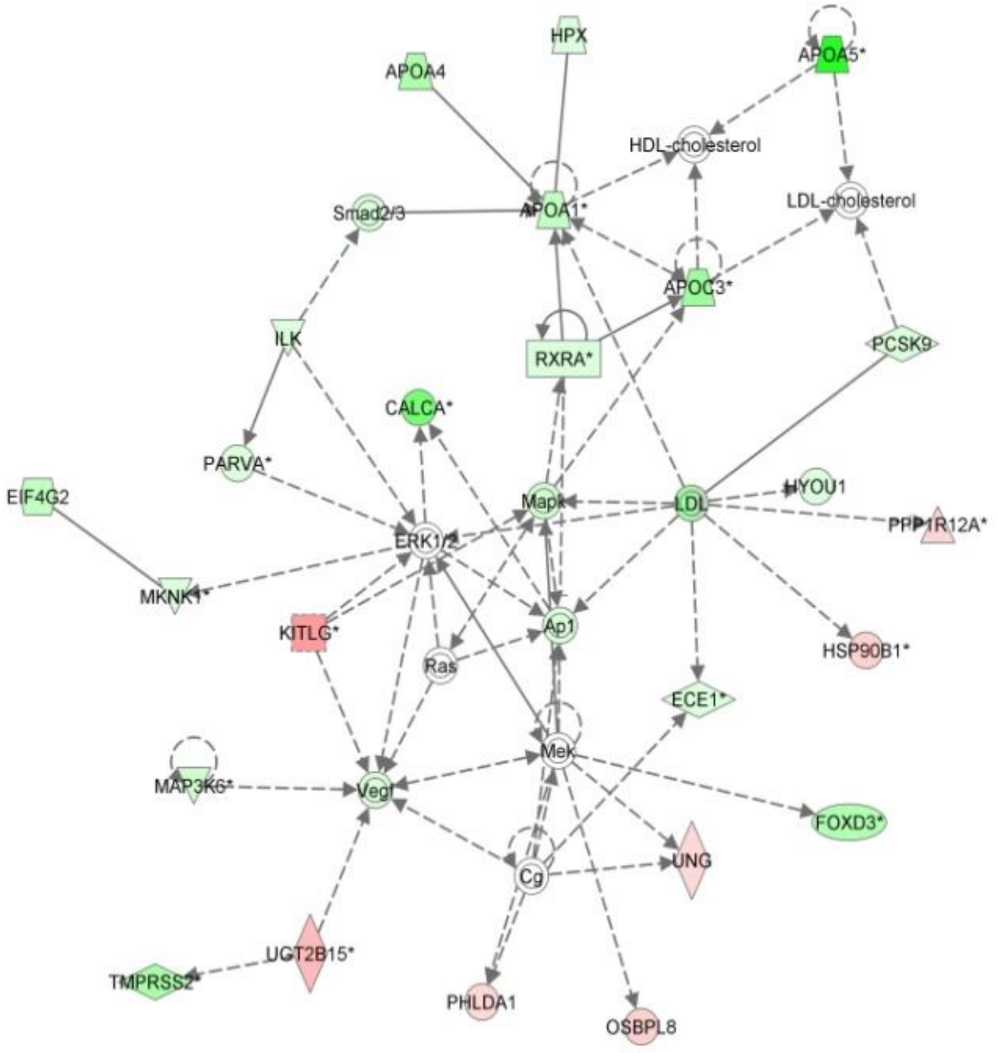

≥50 years/ER+/HER2-

First network

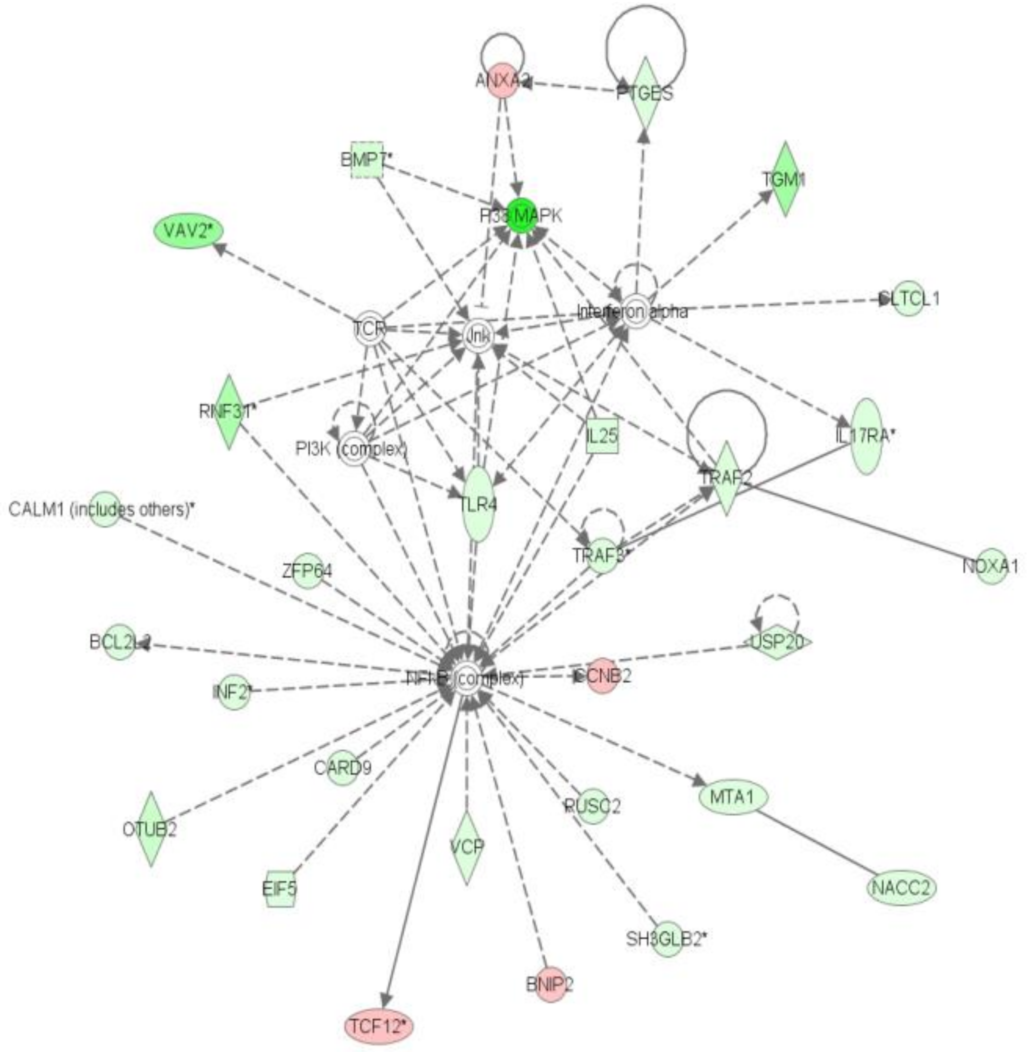

Second network

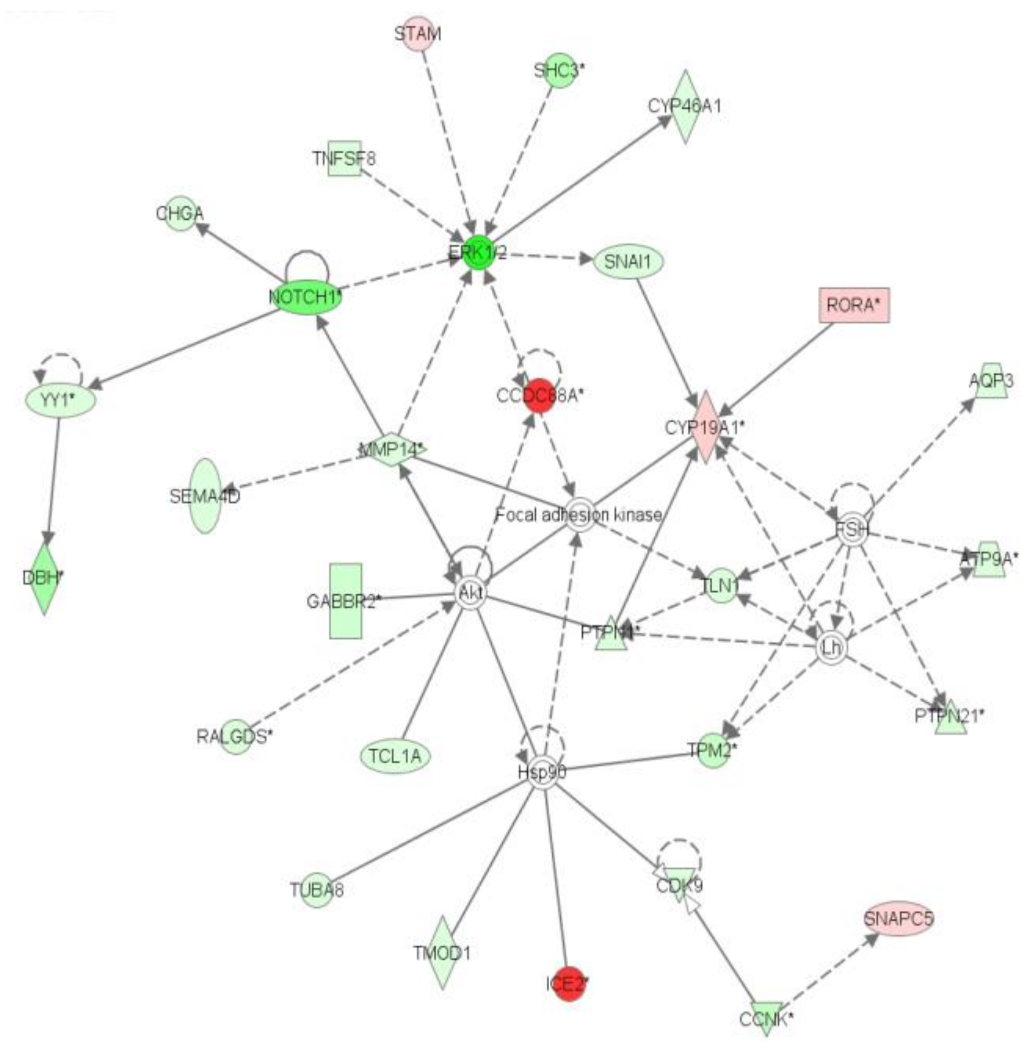

Third network

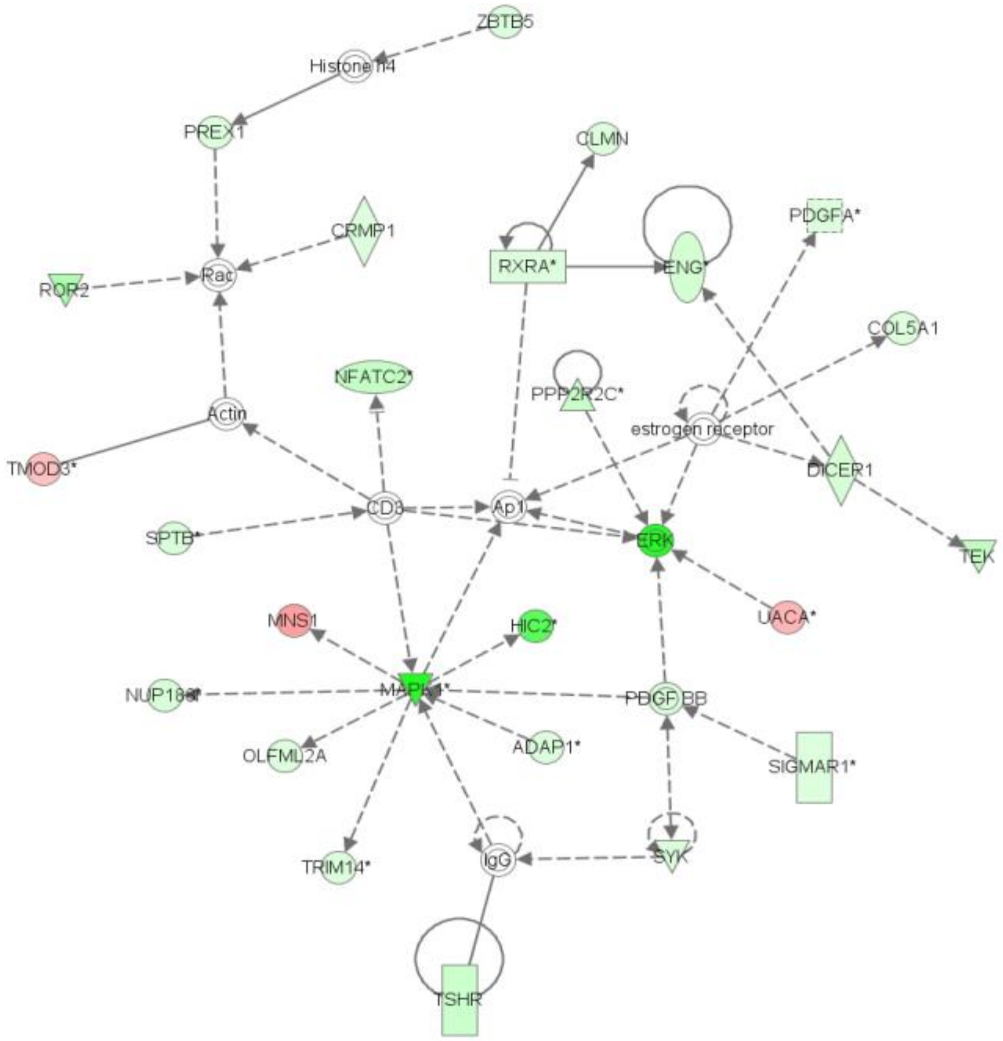

(B)

<50 years/ER-/HER2+

First network

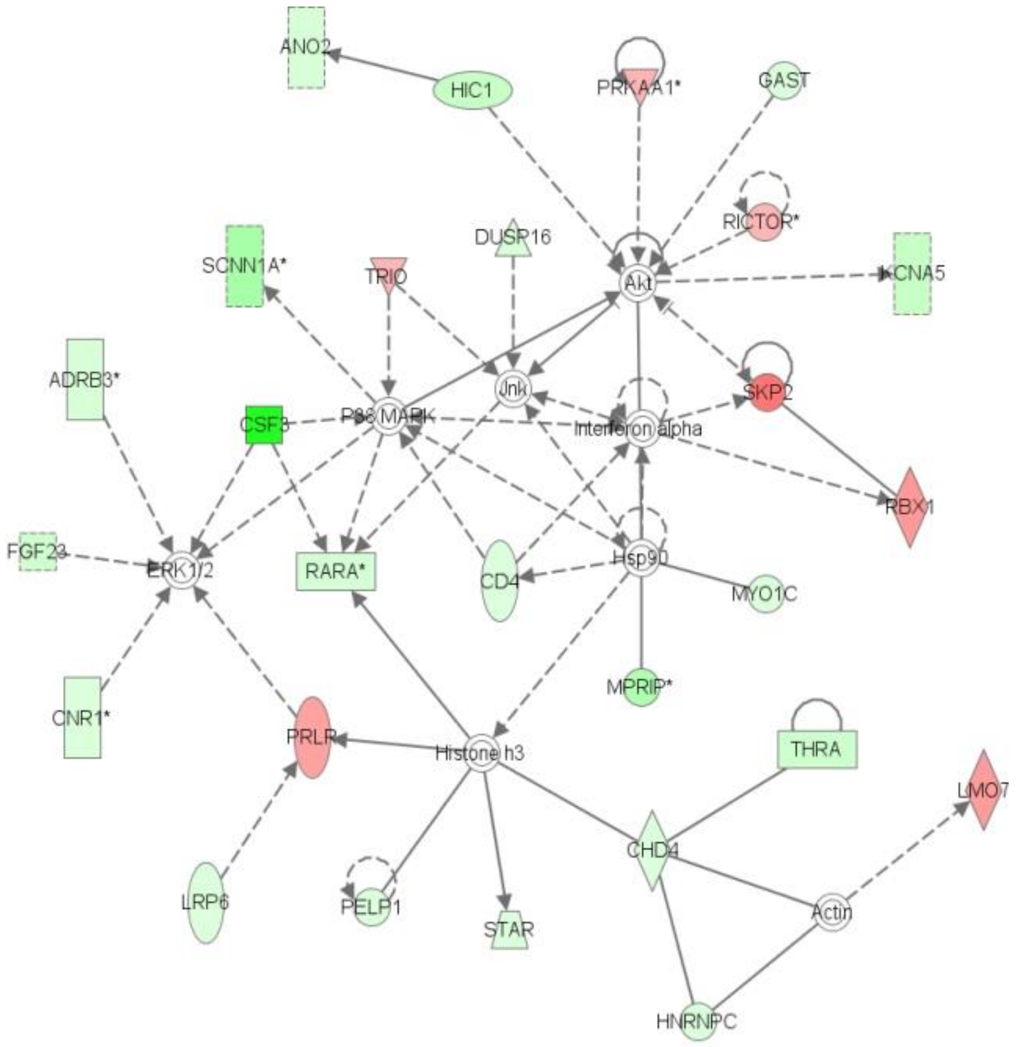

Second network

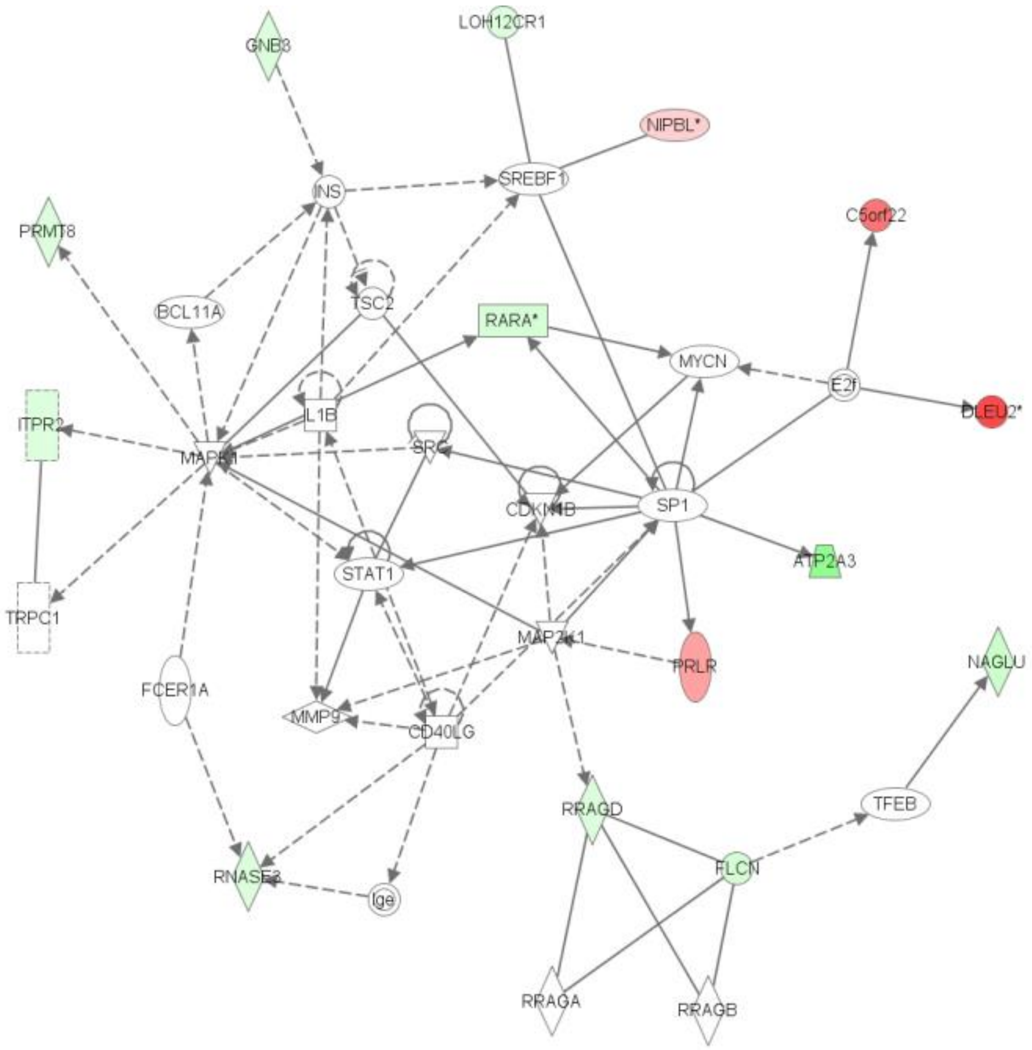

Third network

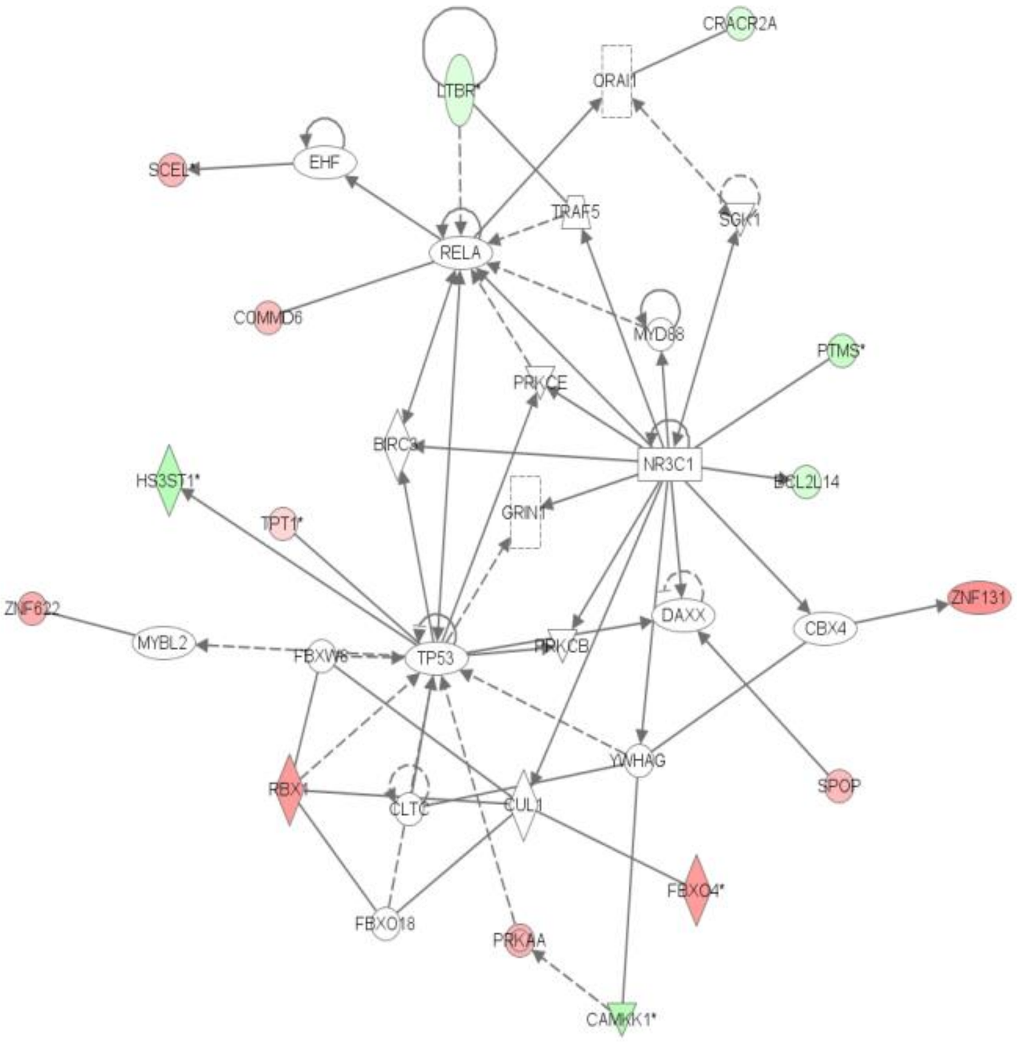

≥50 years/ER-/HER2+

First network

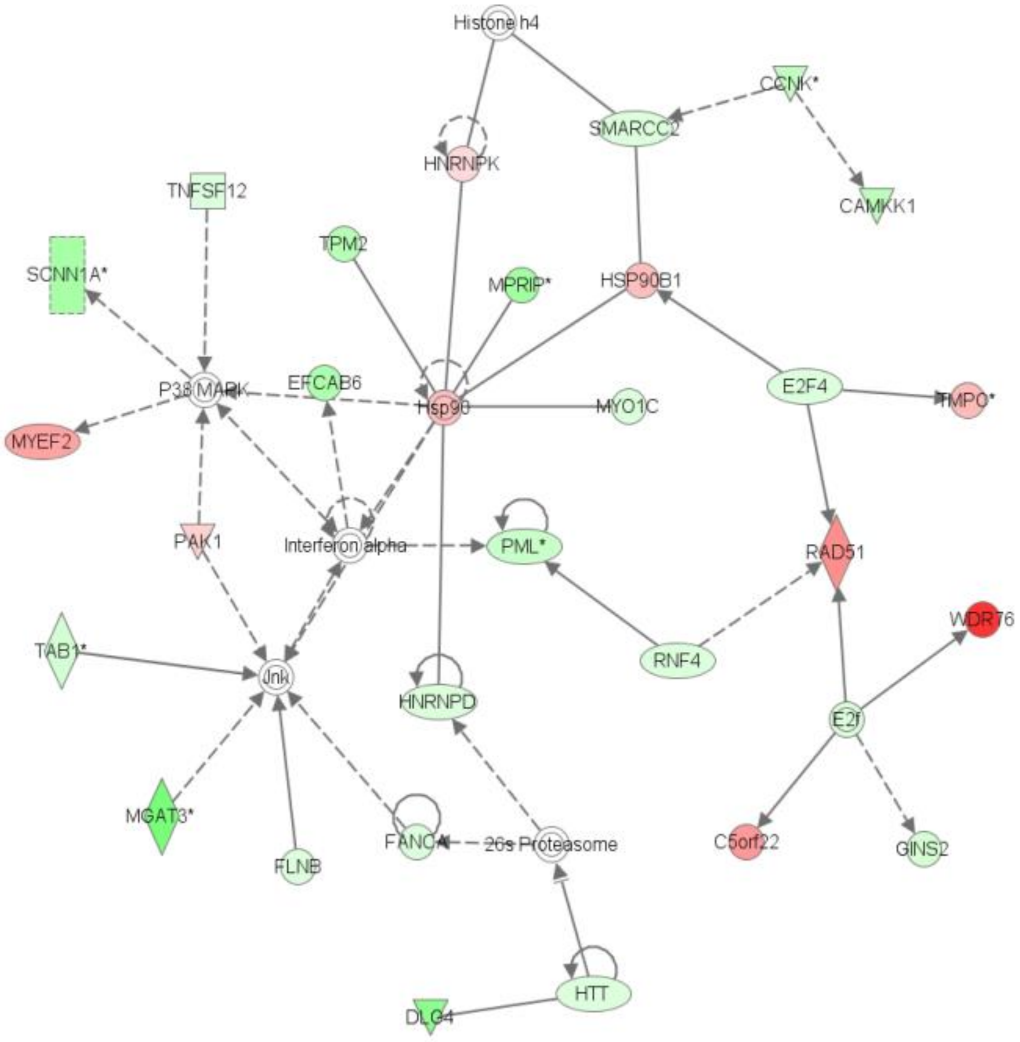

Second network

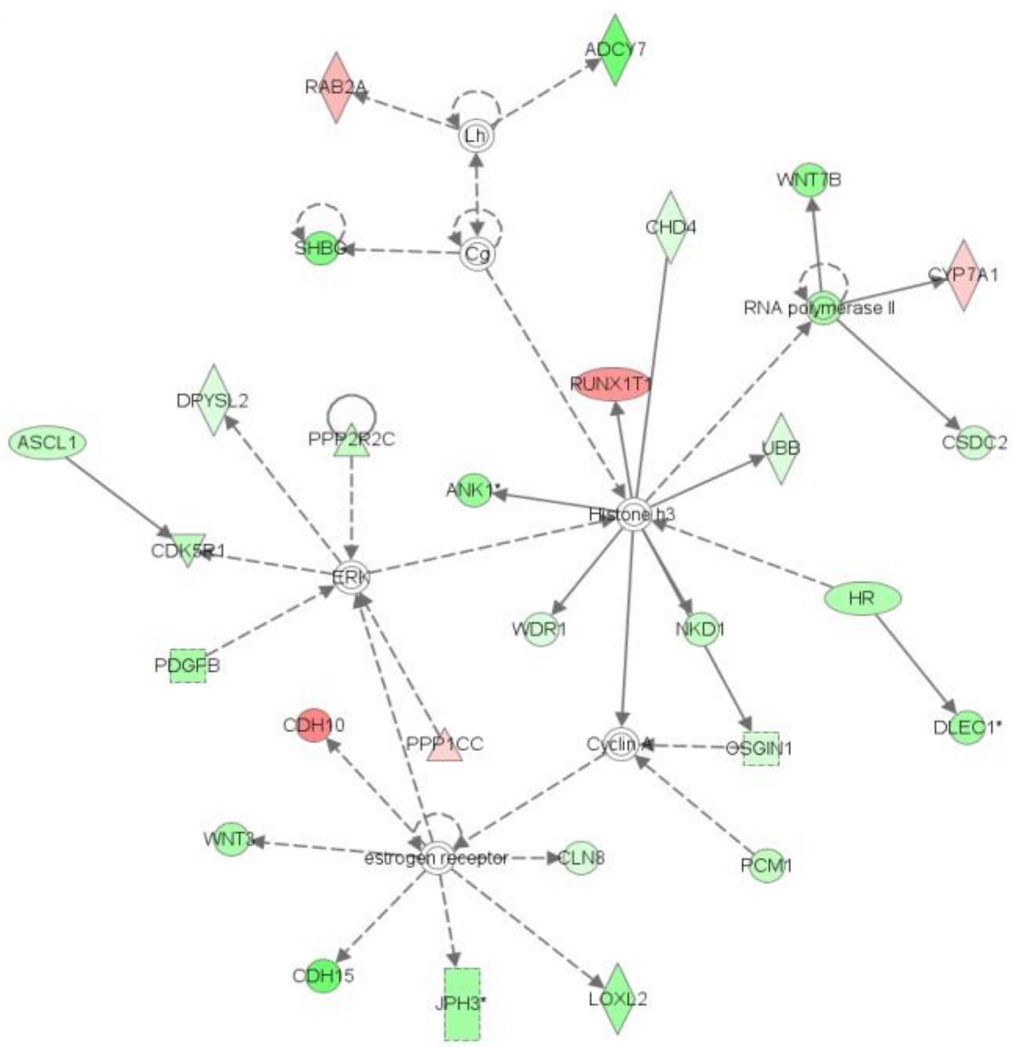

Third network

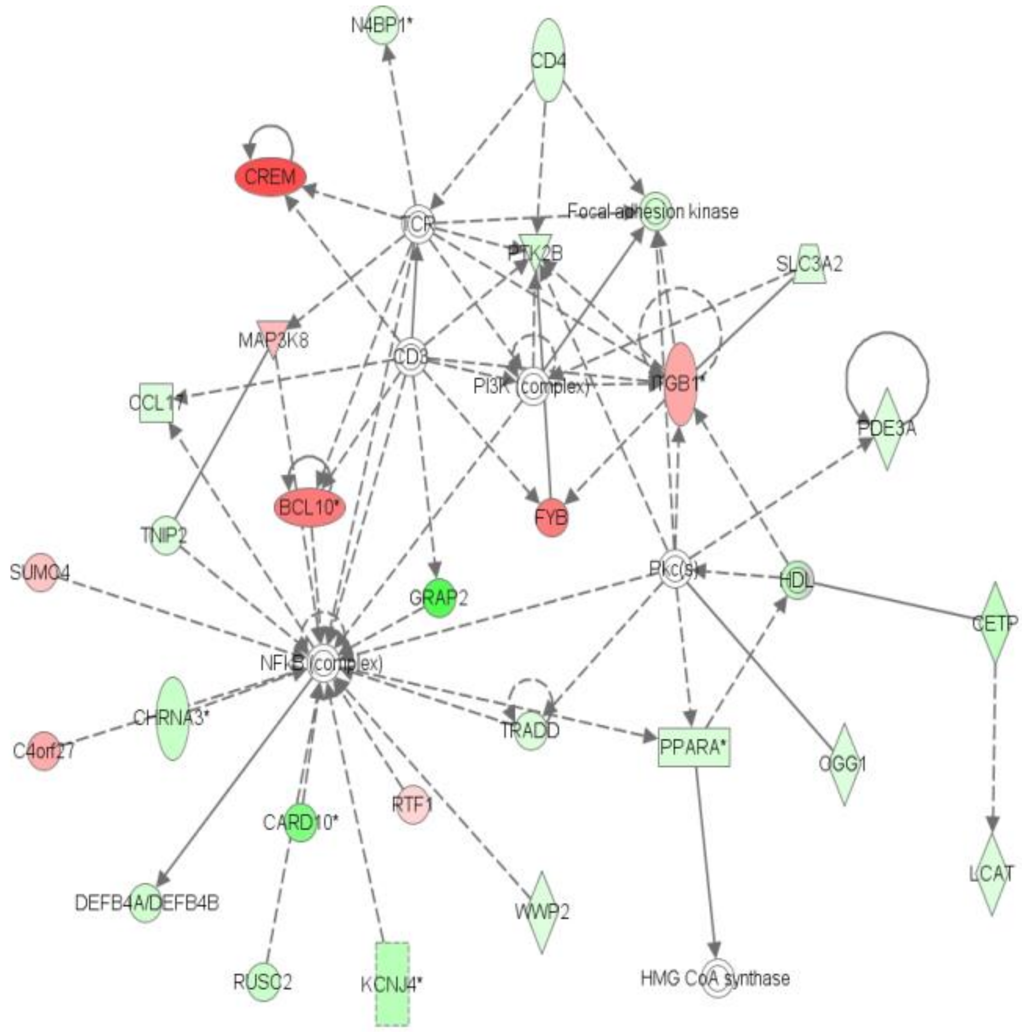

(C)

<50 years/ER-/HER2+

First network

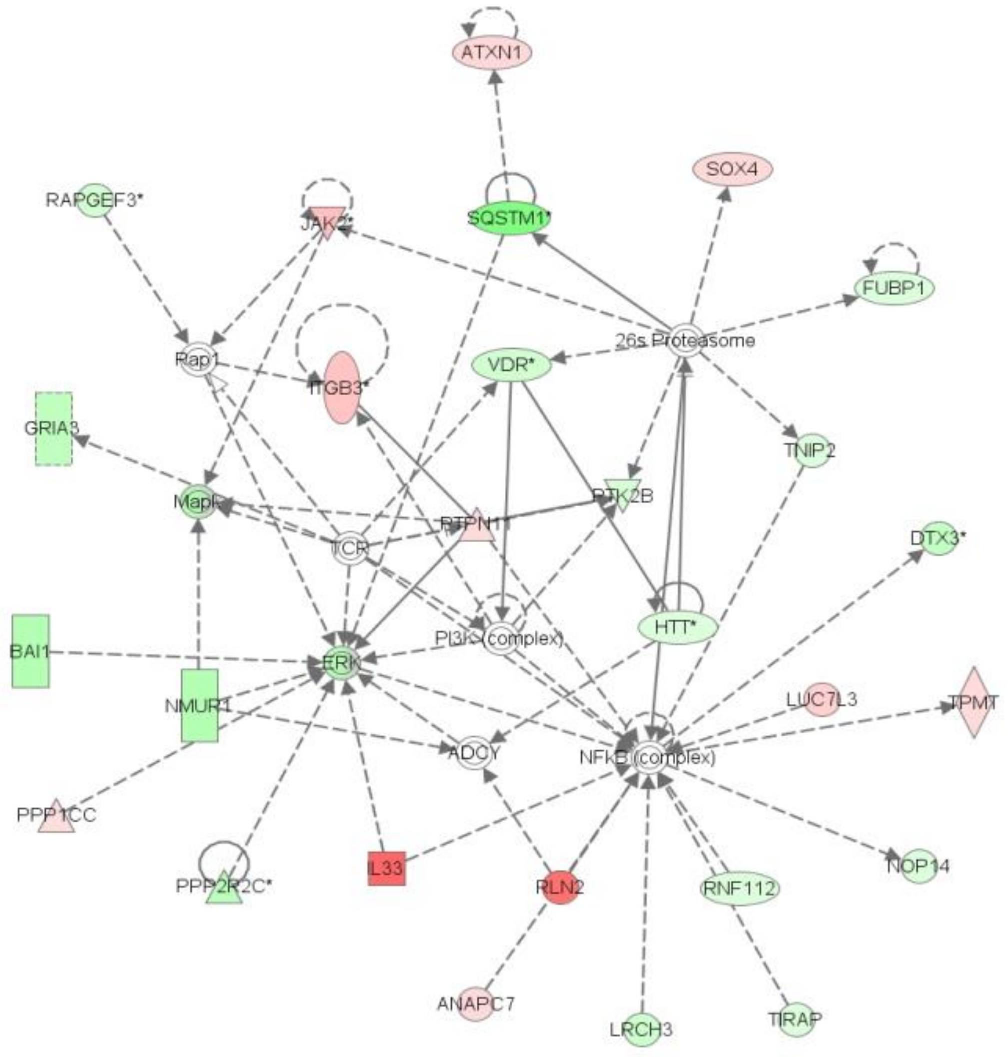

Second network

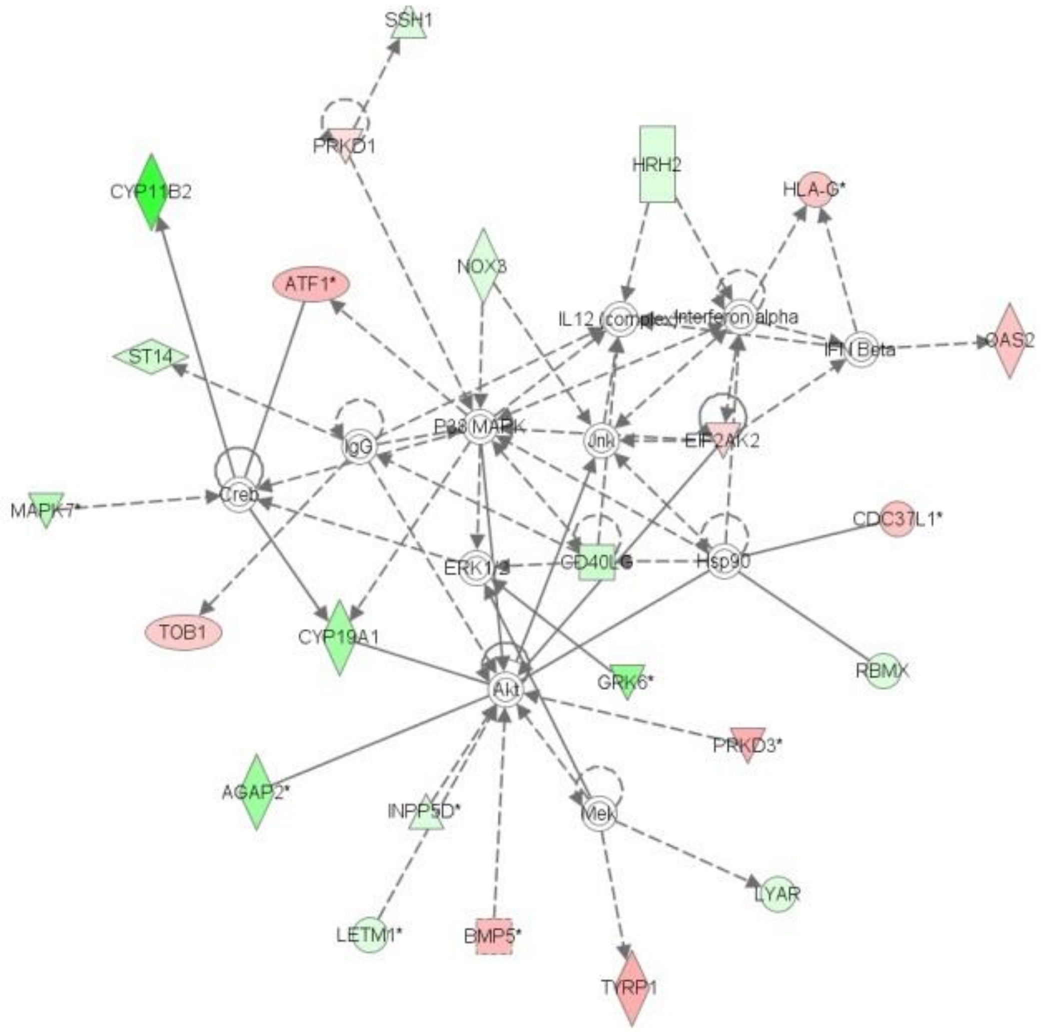

Third network

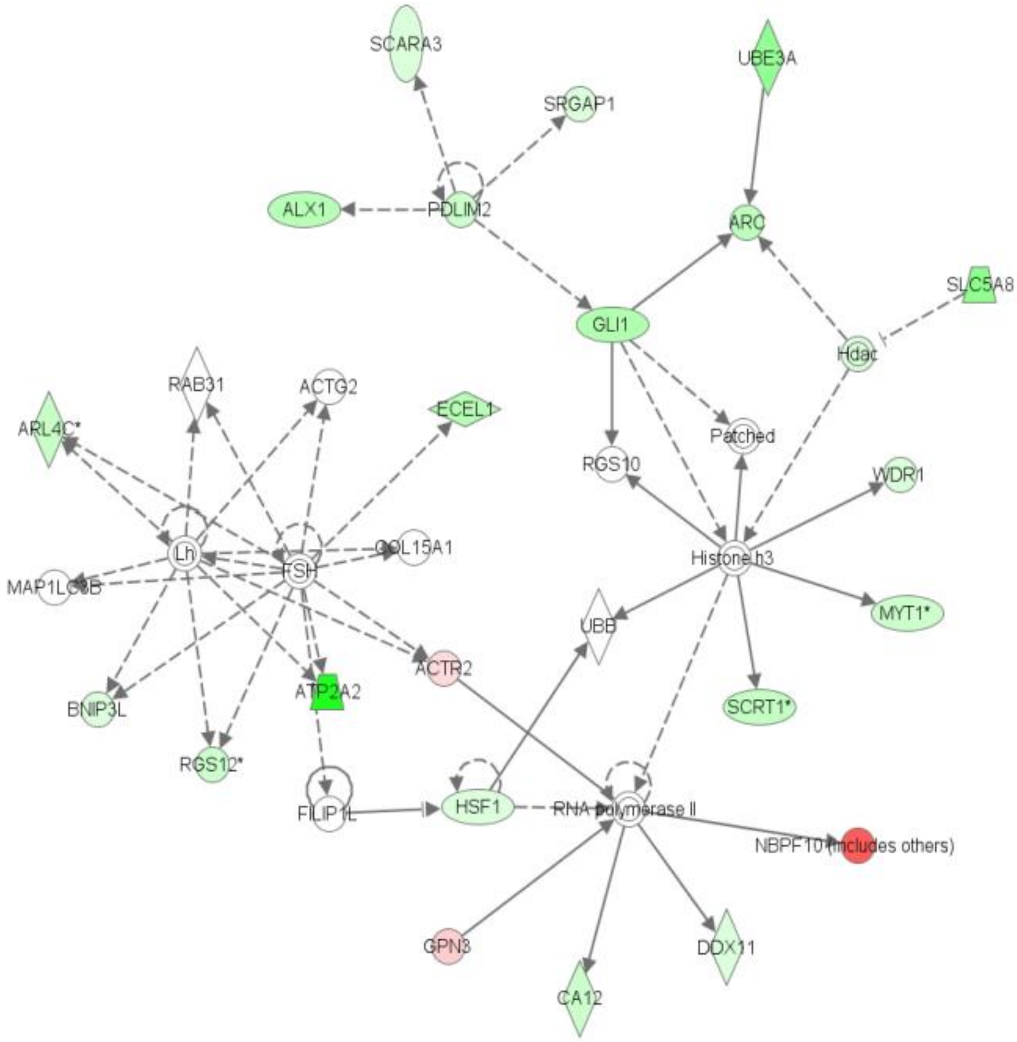

≥50 years/ER-/HER2+

First network

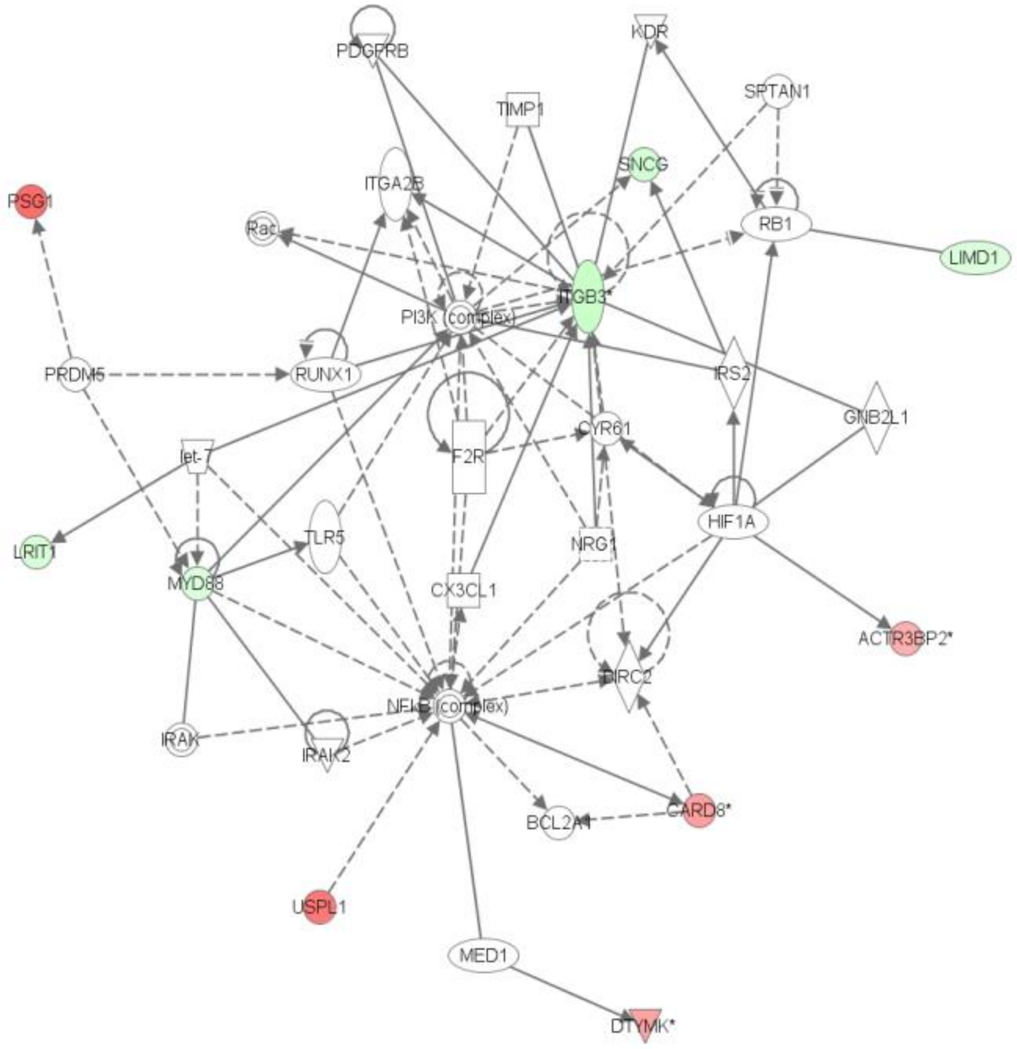

Second network

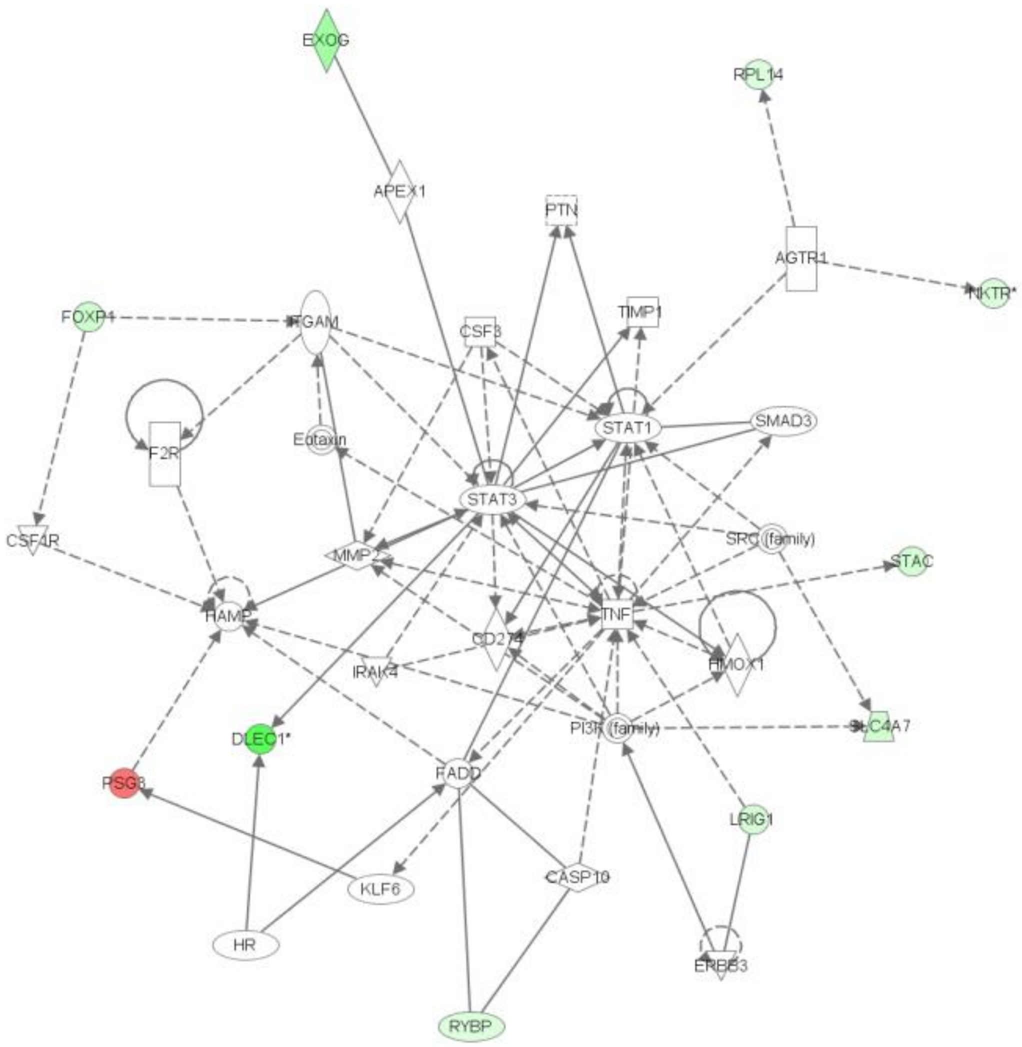

Third network

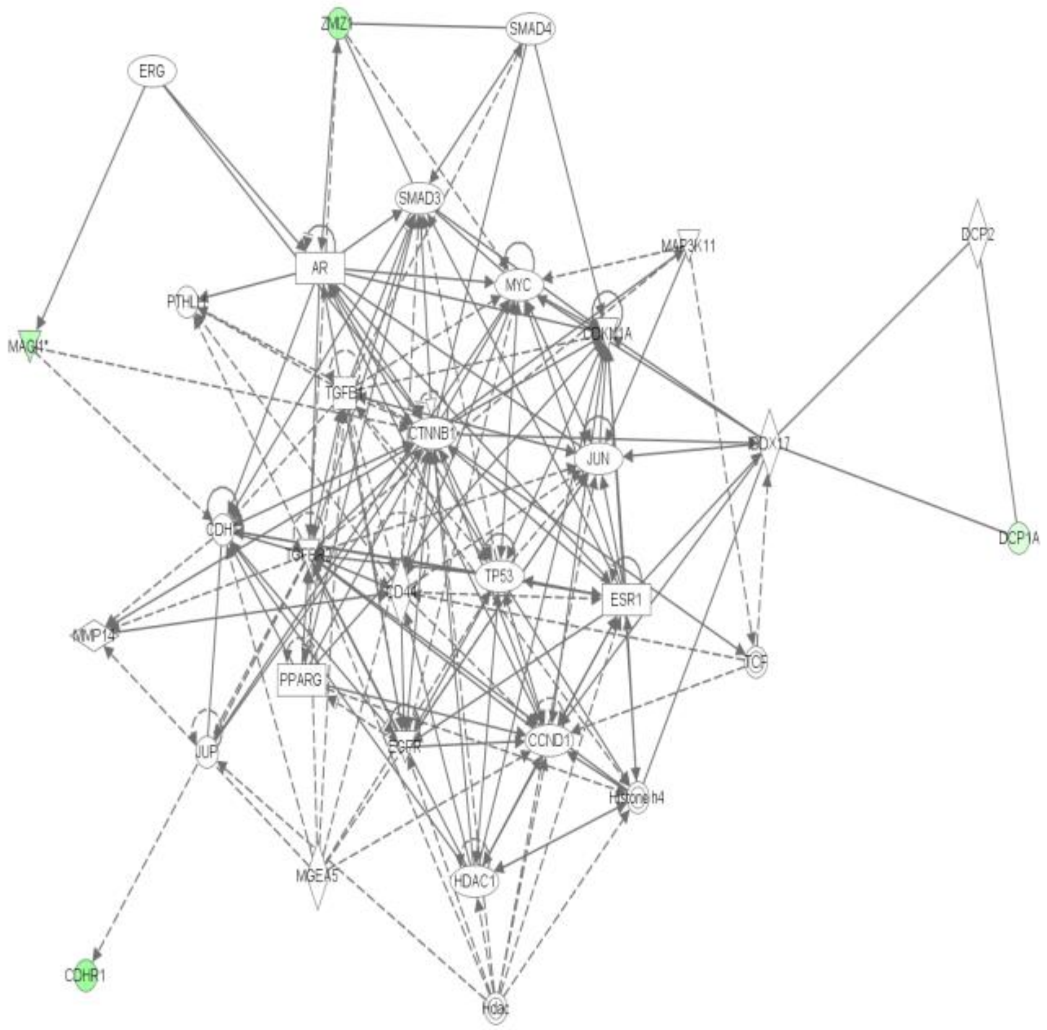

Supplementary Fig. 2 The third major networks of CNV−driven GE differences in age <50 years/ER+/HER2- subgroup between East Asian patients and Western patients (A). The counterpart of the third major network in age ≥50 years/ER+/HER2- (B). Red color denotes up-regulation genes whereas green color denotes down-regulation. The intensity of the red and green colors represents the level of GE difference of breast tumors between East Asian and Western patients. Gray color indicates that the genes were added based on the IPA and were not in the list of identified genes.

(A)

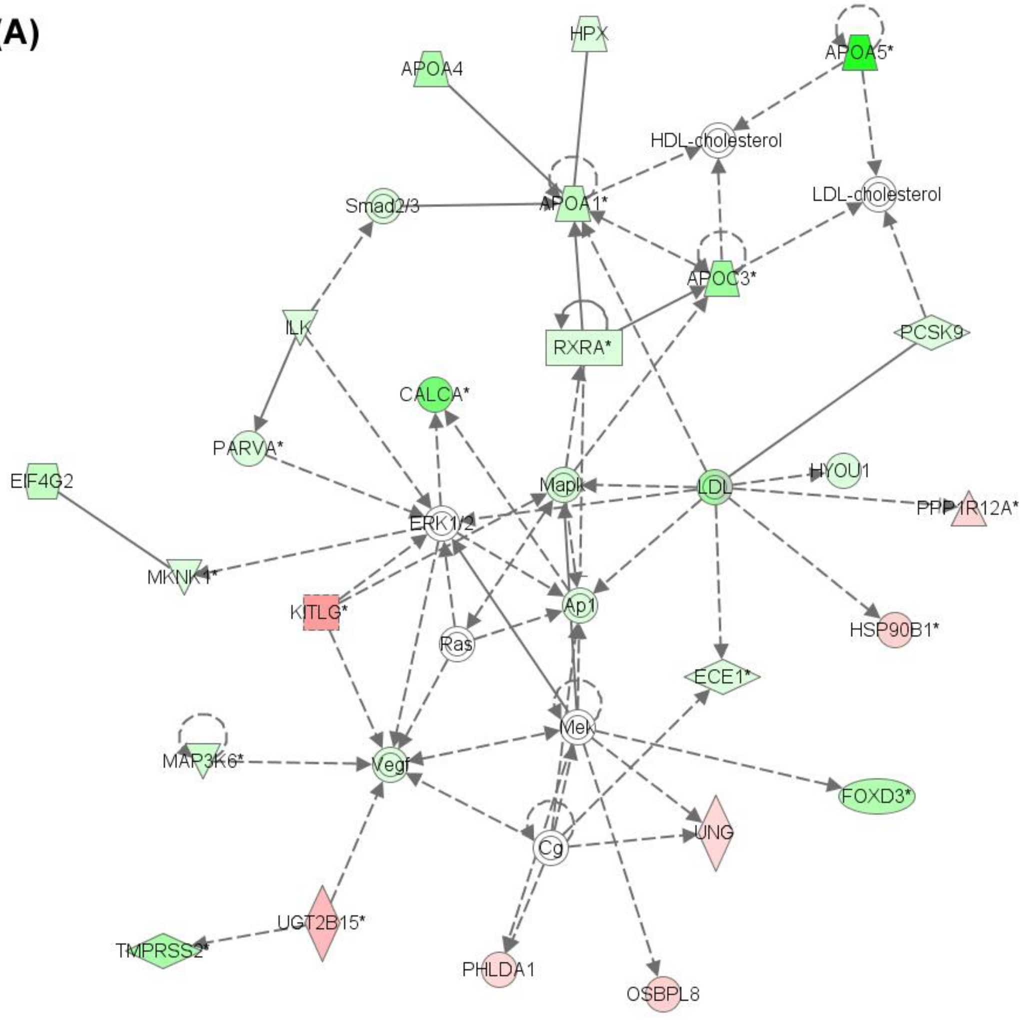

(B)

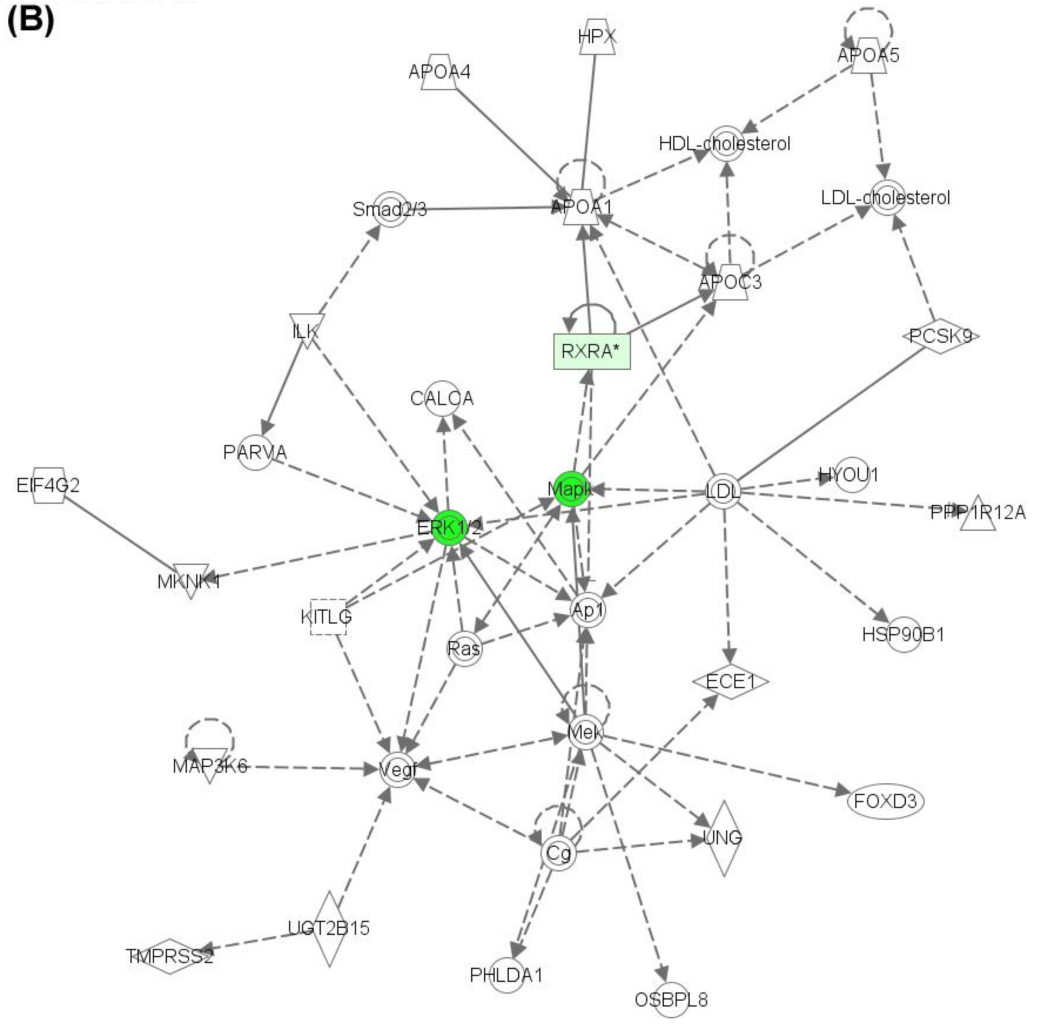
HPX
APOA4
APOA5
HDL-cholesterol
LDL-cholesterol
Smad2/3
APOA1
APOC3
ILK
RXRA*
PCSK9
CALCA
PARVA
EIF4G2
Mapk
LDL
HYOU1
PPP1R12A
ERK1/2
MKNK1
KITLG
Ap1
HSP90B1
Ras
ECE1
Mek
MAP3K6
Vegf
FOXD3
UNG
Cg
UGT2B15
TMPRSS2
PHLDA1
OSBPL8

Supplementary Fig. 3 CNV patterns of breast tumors in age ≥50 years/ER+/HER2- subgroup of NTUH discovery cohort and METABRIC cohort.

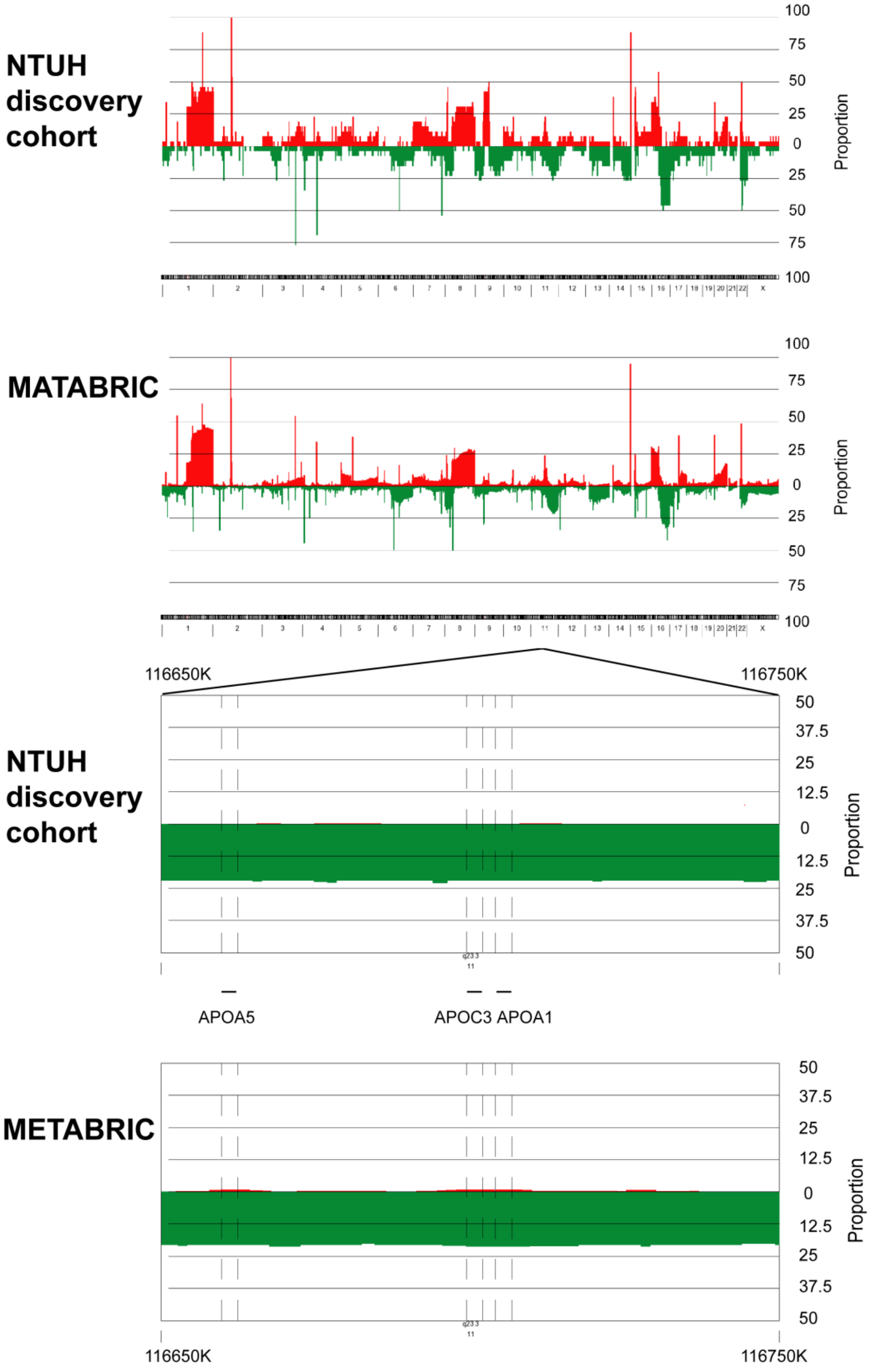

Supplementary Fig. 4 Validation of APOC3 (A) and APOA5 (B) copy number alterations by TaqMan Copy Number Assay

(A) APOC3 The blue and yellow bars represents the copy number levels measured by TagMan Copy Number Assay in cases with copy number normal and loss defined by Affymetrix SNP 6.0 arrays, respectively.

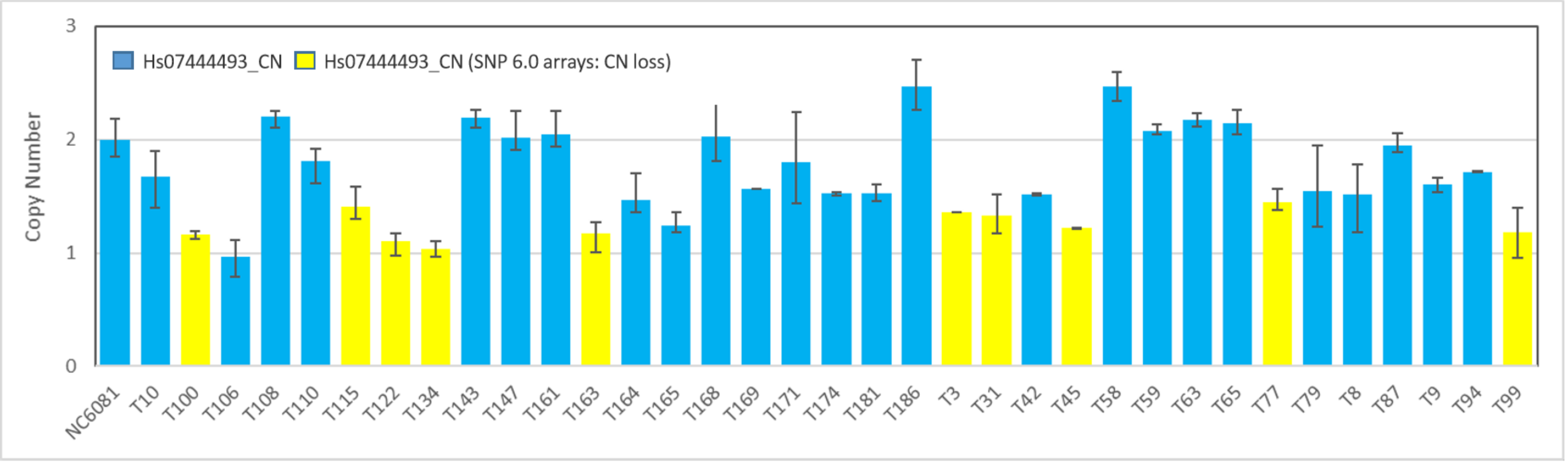

(B) APOA5 The blue and light blue bars represents the copy number levels measured by TagMan Copy Number Assay in cases with copy number normal defined by Affymetrix SNP 6.0 arrays. The orange and light yellow bars represents the copy number levels measured by TagMan Copy Number Assay in cases with copy number loss defined by Affymetrix SNP 6.0 arrays

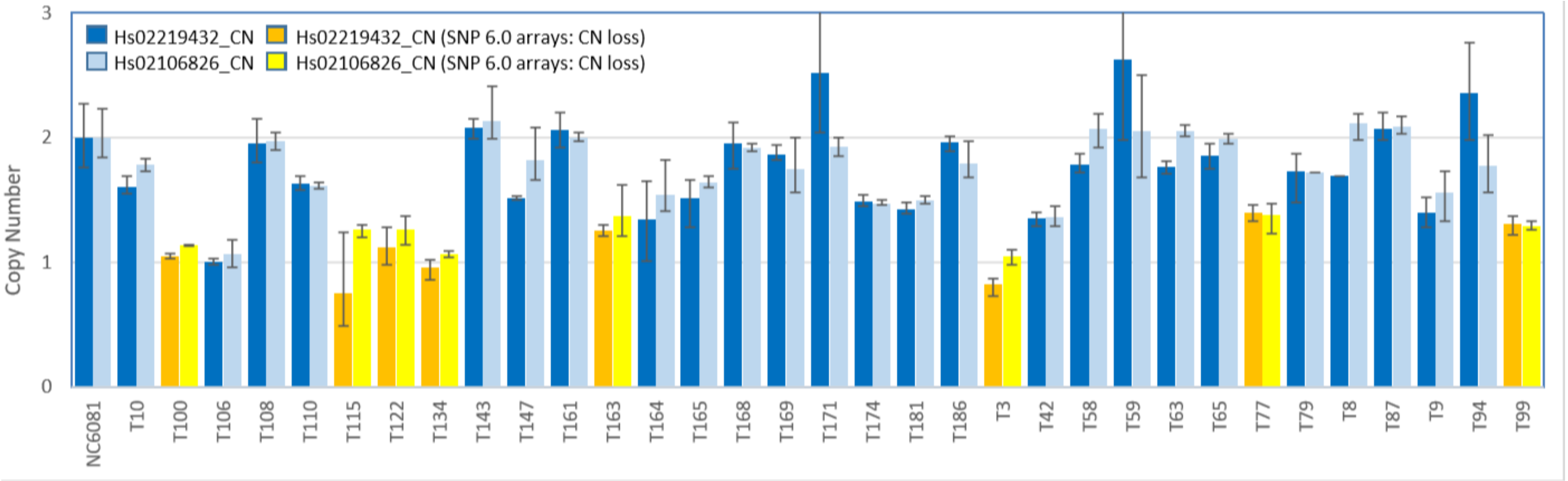

Supplementary Fig. 5 Kaplan–Meier plots of disease-free survival by the *APOA1/C3/A4/A5* copy number loss in NTUH exploratory cohort (A) and in the NUTH validation cohort (B), and breast cancer-specific survival by the *APOA1/C3/A4/A5* copy number loss in METABRIC cohort (C) (unadjusted analysis)

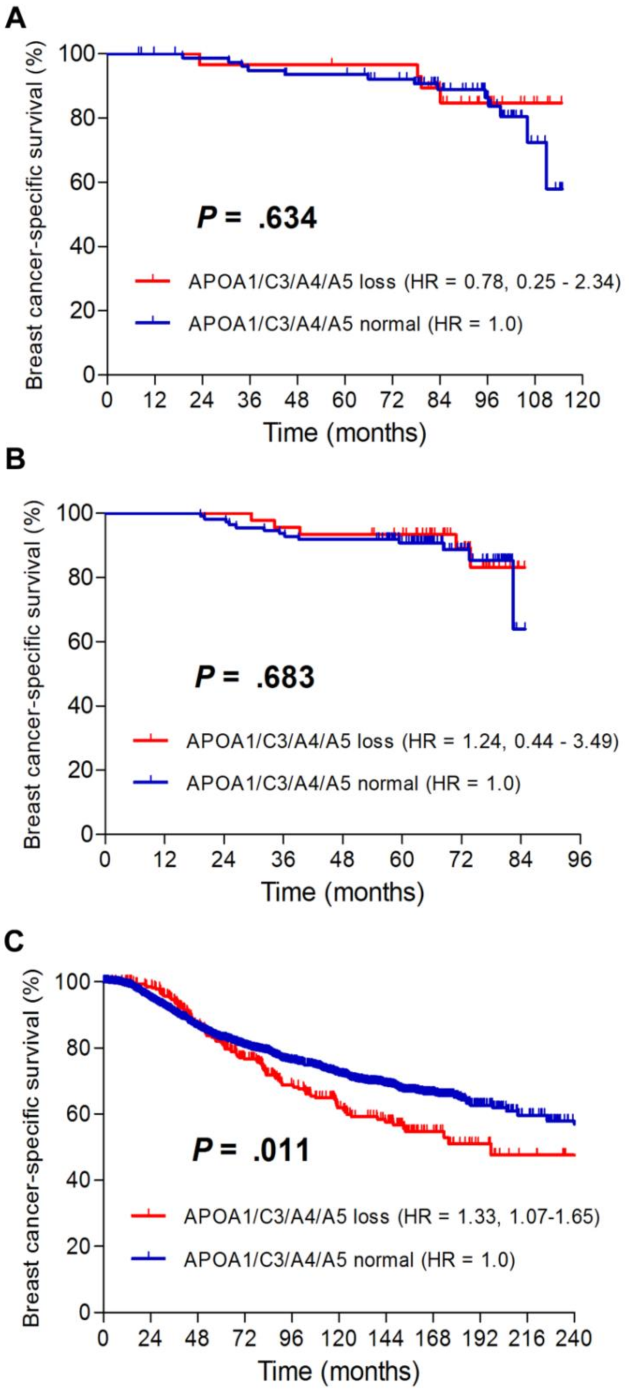

Supplement: Supplementary file 1 — Supplementary Information [file 41523_2021_299_MOESM1_ESM.pdf]
