# Supplementary material for: High prevalence of APOA1/C3/A4/A5 alterations in luminal breast cancers among young women in East Asia
Source: NPJ Breast Cancer. 2021 Jul 5;7:88. doi: 10.1038/s41523-021-00299-5 (PMC8257799; doi:10.1038/s41523-021-00299-5)
Supplement: Supplementary file 2 — Reporting Summary [file 41523_2021_299_MOESM2_ESM.pdf]

nature research

Corresponding author(s): Chiun-Sheng Huang, NPJBCANCER-00856R1

Last updated by author(s): Jun 8, 2021

Reporting Summary

Nature Research wishes to improve the reproducibility of the work that we publish. This form provides structure for consistency and transparency in reporting. For further information on Nature Research policies, see our Editorial Policies and the Editorial Policy Checklist.

Statistics

For all statistical analyses, confirm that the following items are present in the figure legend, table legend, main text, or Methods section.

n/a	Confirmed	
☐	☒	The exact sample size (*n*) for each experimental group/condition, given as a discrete number and unit of measurement
☐	☒	A statement on whether measurements were taken from distinct samples or whether the same sample was measured repeatedly
☐	☒	The statistical test(s) used AND whether they are one- or two-sided<br>*Only common tests should be described solely by name; describe more complex techniques in the Methods section.*
☐	☒	A description of all covariates tested
☒	☐	A description of any assumptions or corrections, such as tests of normality and adjustment for multiple comparisons
☐	☒	A full description of the statistical parameters including central tendency (e.g. means) or other basic estimates (e.g. regression coefficient) AND variation (e.g. standard deviation) or associated estimates of uncertainty (e.g. confidence intervals)
☒	☐	For null hypothesis testing, the test statistic (e.g. *F*, *t*, *r*) with confidence intervals, effect sizes, degrees of freedom and *P* value noted<br>*Give P values as exact values whenever suitable.*
☒	☐	For Bayesian analysis, information on the choice of priors and Markov chain Monte Carlo settings
☒	☐	For hierarchical and complex designs, identification of the appropriate level for tests and full reporting of outcomes
☒	☐	Estimates of effect sizes (e.g. Cohen's *d*, Pearson's *r*), indicating how they were calculated

Our web collection on statistics for biologists contains articles on many of the points above.

Software and code

Policy information about availability of computer code

Data collection	No software was used.
Data analysis	Partek Genomic Suite 6.5 (Partek, St Louis, Missouri, USA) was used for analysis of copy number variations.

For manuscripts utilizing custom algorithms or software that are central to the research but not yet described in published literature, software must be made available to editors and reviewers. We strongly encourage code deposition in a community repository (e.g. GitHub). See the Nature Research guidelines for submitting code & software for further information.

Data

Policy information about availability of data

All manuscripts must include a data availability statement. This statement should provide the following information, where applicable:

- Accession codes, unique identifiers, or web links for publicly available datasets
- A list of figures that have associated raw data
- A description of any restrictions on data availability

The CNV dataset of the 120 breast tumors in NTUH discovery cohort was designated as the GSE80526 dataset.

Field-specific reporting

Please select the one below that is the best fit for your research. If you are not sure, read the appropriate sections before making your selection.

☒ Life sciences ☐ Behavioural & social sciences ☐ Ecological, evolutionary & environmental sciences

For a reference copy of the document with all sections, see nature.com/documents/nr-reporting-summary-flat.pdf

Life sciences study design

All studies must disclose on these points even when the disclosure is negative.

Sample size	We did not predetermine the sample size. We used two cohorts (discovery cohort and validation cohort) to reduce the random error.
Data exclusions	Patients with invasive breast cancer and signed informed consent were included. We did not set up the exclusion criteria for patient enrollment.
Replication	The robustness of our CNV analysis was supported by a strong correlation of HER2 amplification measured by SNP6.0 analysis with overexpression and/or amplification measured by IHC and FISH.
Randomization	The patients were designated as either National Taiwan University Hospital (NTUH) discovery cohort or the validated cohort according to the quantity of tumor DNA extracted. It is not through randomization.
Blinding	The grouping was based on the quantity of tumor DNA extracted, because the samples with inadequate DNA amount cannot fit the requirement for SNP array assay. Blinding was not used in the present study.

Reporting for specific materials, systems and methods

We require information from authors about some types of materials, experimental systems and methods used in many studies. Here, indicate whether each material, system or method listed is relevant to your study. If you are not sure if a list item applies to your research, read the appropriate section before selecting a response.

Materials & experimental systems

n/a	Involved in the study	
☒	☐	Antibodies
☒	☐	Eukaryotic cell lines
☒	☐	Palaeontology and archaeology
☒	☐	Animals and other organisms
☐	☒	Human research participants
☐	☒	Clinical data
☒	☐	Dual use research of concern

Methods

n/a	Involved in the study	
☒	☐	ChIP-seq
☒	☐	Flow cytometry
☒	☐	MRI-based neuroimaging

Human research participants

Policy information about studies involving human research participants

Population characteristics	The population characteristics included age, stage, grade, positivity of estrogen receptor and progesterone receptor, and overexpression/ amplification of HER2.
Recruitment	We recruited the patients with newly diagnosed breast cancer. We did not have exclusion criteria, and tumor characteristics of the present study was consistent with the demographics of breast cancer in Taiwan. No pre-selection bias is suggested.
Ethics oversight	The study's protocol was approved by the ethics committee of NTUH (200902014R)

Note that full information on the approval of the study protocol must also be provided in the manuscript.

Clinical data

Policy information about clinical studies

All manuscripts should comply with the ICMJE guidelines for publication of clinical research and a completed CONSORT checklist must be included with all submissions.

Clinical trial registration	This is not a clinical trial, so we do not have registration number from ClinicalTrials.gov.
Study protocol	The full study protocol cannot be assessed, because it was deposited in NTUH IRB review system with password protection and it was

Study protocol	made in Chinese.
Data collection	Freshly frozen primary tumors and matched blood samples were collected from patients with breast cancer diagnosed between April 2009 and July 2011 at NTUH, Taiwan. The clinicopathological information of these patients was obtained from electronic medical record.
Outcomes	For survival analysis, we used disease-free survival for NTUH cohorts and used breast cancer-specific survival for METABRIC cohort as the primary outcome.
